# Probing atom dynamics of excited Co-Mo-S nanocrystals in 3D

**DOI:** 10.1038/s41467-021-24857-4

**Published:** 2021-08-18

**Authors:** Fu-Rong Chen, Dirk Van Dyck, Christian Kisielowski, Lars P. Hansen, Bastian Barton, Stig Helveg

**Affiliations:** 1 Department of Materials Science and Engineering, City University of Hong Kong, Kowloon Tong, Hong Kong, SAR; 2 EMAT, University of Antwerp, Antwerp, Belgium; 3 The Molecular Foundry, Lawrence Berkeley National Laboratory, Berkeley, CA USA; 4 Haldor Topsoe A/S, Haldor Topsøes Allé 1, Kgs. Lyngby, Denmark; 5 Center for Visualizing Catalytic Processes (VISION), Department of Physics, Technical University of Denmark, Kgs. Lyngby, Denmark

**Keywords:** Heterogeneous catalysis, Imaging techniques, Transmission electron microscopy, Nanoparticles

## Abstract

Advances in electron microscopy have enabled visualizations of the three-dimensional (3D) atom arrangements in nano-scale objects. The observations are, however, prone to electron-beam-induced object alterations, so tracking of single atoms in space and time becomes key to unravel inherent structures and properties. Here, we introduce an analytical approach to quantitatively account for atom dynamics in 3D atomic-resolution imaging. The approach is showcased for a Co-Mo-S nanocrystal by analysis of time-resolved in-line holograms achieving ~1.5 Å resolution in 3D. The analysis reveals a decay of phase image contrast towards the nanocrystal edges and meta-stable edge motifs with crystallographic dependence. These findings are explained by beam-stimulated vibrations that exceed Debye-Waller factors and cause chemical transformations at catalytically relevant edges. This ability to simultaneously probe atom vibrations and displacements enables a recovery of the pristine Co-Mo-S structure and establishes, in turn, a foundation to understand heterogeneous chemical functionality of nanostructures, surfaces and molecules.

## Abstract (web summary)

The authors introduce an analytical approach for quantitative analysis of 3D atom dynamics during electron microscopy. They image a Co-Mo-S nanocrystal with 1.5 Å resolution, and observe chemical transformations caused by beam-stimulated vibrations.

## Introduction

**Fig. 1:**
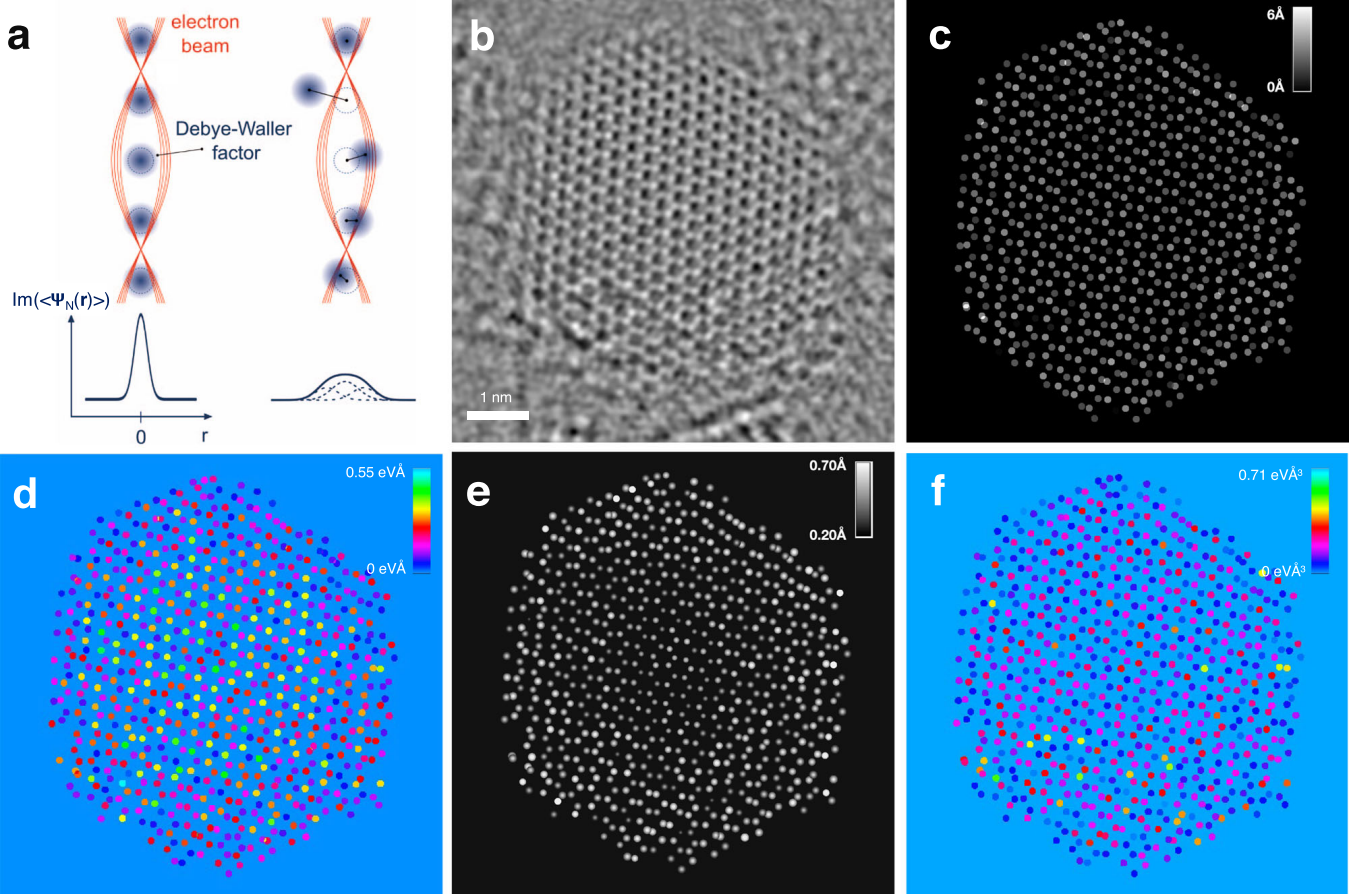
Dynamic analysis of an exit wave. **a** Illustration of the generic model Eq. (1) of the exit wave imaginary part Im(<Ψ_*N*_(**r**)>) from a static column of atoms, modulated by DW factors, and a dynamic column with atom excursions exceeding the DW value. **b**–**f** Benchmark application of model Eq. (1) to the analysis of a Co–Mo–S nanocrystal. **b** The imaginary part of the EW1 of a Co–Mo–S nanocrystal viewed in <001> orientation. **c** Height map showing the atomic column positions along the beam direction with respect to a common image plane as a function of the position in the image plane. **d** *V*/(π*R*^2^) map showing the projected atomic column potentials scaled by the averaged area of the atoms. **e** *R*_*av*_ map showing the spread radius of the atomic columns. **f** *V* map showing the integrated potential of the atomic columns.

It is well understood that material properties are encoded in the choice of elements and their three-dimensional arrangements^1^. This knowledge has driven improvements of transmission electron microscopes, which have reached an ultimate spatial resolution around 50 pm in both focused- and broad-beam modes and provided capabilities for rendering atom positions in three spatial dimensions (3D) by processing images using a variety of mathematical reconstruction methods^2–8^. Traditionally, all reconstruction schemes consider the object as a static structure, which is modulated by a uniform thermal blur described by classical Debye–Waller (DW) factors. The 3D atomic structure determination relies currently on electron microscopy conducted at high electron doses corresponding to 10^5^ e^−^/Å^2^ and 500 e^−^/Å^2^ to detect a single C or Au atom, respectively, in broad-beam mode^9^. Under such illumination conditions, excitations can cause displacements of atoms from their equilibrium positions that by far exceed the thermal vibrations predicted by DW factors^10^ (Fig. 1a).

In fact, nano-scale objects have functional properties that strongly depend on the surface atoms. The surface atoms have reduced coordination, which consequently makes them more prone—compared to bulk atoms—to alterations induced by any stimuli, including gas/liquid environments, heat, electromagnetic fields as well as electron beams^11–16^. 3D atomic-resolution images have uncovered atom dynamics under an assumed uniform excitation^11,12^ and 2D projected atomic-resolution images have revealed atom displacements at nanoparticle surfaces^15^, even with a site-dependency^16^. Thus, the dynamic behavior of nano-scale objects is generally modulated in space and time and expected to heterogenize the electron microscopy image intensities and contrast blurring. To account for such image variations, 3D atomic-resolution electron microscopy is needed with a larger temporal resolution ranging from seconds^11,12^, characteristic of chemical kinetics, toward femto-seconds, characteristic of electronic processes^17^. Advancing such 3D atomic-resolution imaging with time-resolution meets significant challenges including the development of suitable tools to analyze the dynamic behavior of atoms at any chosen timescale^10^.

Here, we expand existing experimental and theoretical capabilities by first introducing an analytical approach to extract all spatiotemporal information encoded into transmission electron microscopy (TEM) data up to the limits of the counting statistics. By reconstruction of the electron wave exiting the specimen, sensitivity to the electrostatic potential of single atoms is achieved despite using reduced electron dose rates and doses. Thus, the approach becomes generally applicable for atomic-resolution electron microscopy of nano-scale objects and electron-beam-sensitive materials. Subsequently, the approach is merited by the dynamic analysis of Co–Mo–S nanocrystals in 3D.

## Results and discussion

### Analytical model for the exit wave of a dynamic crystalline object

The most informative fingerprint of the atomic structure is contained in the electron exit wave. It can be reconstructed by either off-axis^18^ or in-line holography^19^. For the latter, a focal image series is recorded of the specimen using the TEM broad-beam mode, which in turn offers the largest signal with the fewest scattered electrons. The exit wave of a thin object in zone-axis orientation is modeled by the channeling theory^20,21^, which describes how electrons are trapped in atomic columns parallel to the electron beam. The exit wave of an atomic column can be expressed in a first-order approximation as follows (see Supplementary Methods):

1 $$ < {\varPsi }_{{{{{\rm{N}}}}}}({{{{{\bf{r}}}}}}) > ={{{{{\rm{i}}}}}}{\alpha }{Z}{a}^{2}({{{{{\rm{V}}}}}}_{{{{{\rm{eff}}}}}}({{{{{\bf{r}}}}}}))={{{{{\rm{i}}}}}}{\alpha }{Z}{a}^{2}({{{{{\rm{V}}}}}}_{{{{{\rm{p}}}}}}({{{{{\bf{r}}}}}})\otimes {{{{{\rm{b}}}}}}({{{{{\bf{r}}}}}})\otimes {{{{{\rm{P}}}}}}({{{{{\bf{r}}}}}}))$$

Here, **r** is the image plane vector, < > time averaging, α a constant for all atoms, *Z* the projected atomic number of the column, *a* the atom radius, V_eff_(**r**) the effective potential, which is composed of V_p_(**r**) the projected electrostatic potential of the specimen, b(**r**) the normalized thermal distribution of atom positions, and P(**r**) the normalized point-spread function of the microscope covering incoherent aberrations remaining after exit wave restoration, and ⊗ the convolution operator. The above expression for the exit wave is normalized to the vacuum wave outside the object. Moreover, the channeling model is applicable to samples as thin as single atoms^20,21^ and the expression Eq. 1 is accurate to better than 16% for thin samples with a projected *Z* < 200 for all the atoms in an atomic column (Supplementary Methods). Since V_p_(**r**), b(**r**) and P(**r**) are normalized functions that all peak at the equilibrium column position *r* = 0, one finds

2 $${\int} {{{{{\rm{Im}}}}}}( < {\varPsi }_{{{{{{\rm{N}}}}}}}({{{{{\bf{r}}}}}}) > ){{{{{\rm{d}}}}}}{{{{{\bf{r}}}}}}={V}\,{{{{{\rm{with}}}}}}\,{V}={{\alpha }}{Z}{{a}}^{2}$$

with *V* being the integrated exit wave imaginary part of the single-column (in units of eV Å^3^). Furthermore, Im(<Ψ_N_(**r**)>) peaks at the atomic column positions (Fig. 1a) and has an integral proportional to the total *Z* value of the atoms in the column. In general, the intensity profile Eq. (1) is set by the elements, their location and their dynamic behavior as well as by the microscope parameters. Thus, in case of correction for coherent image aberrations, the exit wave intensity will be blurred by the electron scattering cross sections, atom vibrations and incoherent lens aberrations. The blur can result in intensity overlap among neighboring atomic columns in the exit wave, which can hamper the assignment of image pixels to the proper atomic column (Fig. 1a). To improve the accuracy of determining *V*, the full set of pixels that can unambiguously be associated with an atomic column is therefore modeled by a Gaussian blurring function (see Supplementary Methods), i.e.:

3 $${{{{{\rm{Im}}}}}}( < {\varPsi }_{{{{{{\rm{N}}}}}}}({{{{{\bf{r}}}}}}) > )={V^{\prime}}\exp (-{{r}}^{2}/{{R}}^{2})$$

where *V*’ (in units eV Å) equals *V*/(π*R*^2^) and is the projected potential scaled by the averaged area covered by the smeared atoms, and *R* is the radially symmetric spread radius describing the exit wave blurring, i.e.:

4 $${R}^{2}={{R}_{s}}^{2}+{{R}_{v}}^{2}+{{R}_{a}}^{2}$$

with *R*_*s*_, *R*_*v*_, and *R*_*a*_ corresponding to the blurring radius of electron scattering cross sections (s), atom vibrations (v), and incoherent lens aberrations (a), respectively. Thus, the analytical approach extends the static channeling theory^20,21^ of the exit wave by including spatiotemporal dynamic behavior of the atoms described by just two physical object parameters, *V’* and *R*. Specifically, the model includes unrestricted vibrational blur, *R*_*v*_, that can exhibit spatial and temporal dependency and that can exceed usual DW factors depending on the detailed balance between electron-stimulated excitations and relaxations of the object under electron illumination. Importantly, the integrated value *V* is more robust with regard to atom vibrations as compared to the peak value *V*′, which earlier 3D atomic-resolution analysis methods relied on.

### Imaging atom dynamics of a Co–Mo–S nanocrystal in 3D

The analytical model is applicable to analyzing exit waves of any 3D nanomaterial. As an ideal benchmark system, the present analysis focuses on single-layer MoS_2_ nanocrystals with edge-attached Co atoms on a graphitic support. In <001> orientation, the MoS_2_ basal plane exposes of a uniform pattern of one Mo (1Mo) and two S (2S) atomic columns,  which facilitates an unambiguous distinction between atom type and occupancy as well as three-dimensional positions. Moreover, the (001) basal plane is terminated by distinctly different low-indexed {100} edges with unique catalytic properties^22^. The one or two atom-wide edge structures require single-atom-sensitive TEM for a detailed examination^23–25^. However, the reduced coordination makes the edges prone to alterations at cumulative electron doses around 4 × 10^5^ e^−^Å^−2^ delivered at rates of 5 × 10^7^ e^−^Å^−2^s^−1^ in the focused-beam mode^26,27^ or 5 × 10^4^ e^−^Å^−2^ and 300 e^−^Å^−2^ s^−1^ in the broad-beam mode^19^. To monitor such dynamic edge structures, the Co–Mo–S nanocrystals were examined using the TEAM 0.5 transmission electron microscope^19^. The microscope was operated with a primary electron energy of 50 keV in broad-beam mode with an estimated ~1.4 Å information limit, which suffices to resolve the projected Mo–S dumbbell distance of 1.8 Å. The low electron energy suppresses a knock-on displacement of S and Mo atoms from the basal plane and enhances inelastic scattering contributions (“Methods”)^28–30^. The electron beam therefore excites atoms in the nanocrystal with a dynamic response that is expected to depend intimately on the electron dose rate^30–32^. Thus, using low electron dose rates of ca. 89 and 290 e^−^Å^−2^s^−1^, noise-dominated images are recorded in focal series corresponding to a total dose of ca. 3560 and 6380 e^−^Å^−2^, respectively, and with sufficient contrast to restore five successive exit waves of a Co–Mo–S nanocrystal in <001> orientation (“Methods”, Supplementary Figs. 1, 2).

The exit waves reveal that the Co-Mo-S nanocrystal spans a uniform single-layer MoS_2_ plane consisting of hexagonally arranged dumbbells including 1Mo and 2S atomic columns (Fig. 1b and Supplementary Figs. 1, 2)^19^. Their exact lateral positions in the Im(<Ψ_N_(**r**)>) image are determined by fitting a Gaussian function to each peak with sub-pixel precision, whereas their locations along the beam direction are measured by a propagation of intensities followed by a refinement using the Big-Bang scheme^5,7^ (“Methods”). These methods enable the column heights to be determined with high precision. In fact, Fig. 1c and Supplementary Fig. 3 show the atomic column positions along the beam direction and reveal repetitive measurements of the distinct heights within the metal (Mo and edge Co) and S sub-lattices at separations around 1.5 Å consistent with the single-layer of the established 2H-MoS_2_ crystal structure. Thus, the atomic column positions are now measured better than ~1.5 Å in 3D in spite of using single projections only. Moreover, the exit wave values span an almost vertical line in the complex Argand plot (Supplementary Fig. 4) justifying the thin sample approximation (Eqs. 1–3 and Supplementary Methods).

To quantify *V*′ *a*nd *R*, the column intensity profiles are fitted by Eq. 3 (“Methods”). The parameters are presented in maps to display their spatial heterogeneity (Fig. 1d, e). Remarkably, the *V*′ values tend to decrease from the center toward the edge of the nanocrystal (Fig. 1d and Supplementary Fig. 5). Unfortunately, the decrease by 40–50% is sufficiently large to compromise the chemical information encoded in the potential. Moreover, R is represented by the average radius *R*_av_ of the atomic column (Eq. 4 and “Methods”). The *R*_av_ is about 50 pm in the bulk of the nanocrystal and increases towards the edge by another ~10 pm (Fig. 1e and Supplementary Fig. 6a). With an atomic potential radius *R*_*s*_ ~ 30 pm^10^, a vibrational blur in the bulk reaches, according to Eq. 4, up to *R*_*v*_ ~ 40 pm that exceeds typical DW factors (~8 pm at room temperature) under the chosen illumination conditions. This column broadening reflects the anisotropy of the crystal structure (Supplementary Fig. 6), which makes it distinctly different from any optical instability of the electromagnetic lenses (*R*_*a*_) that is unrelated to sample properties and affect the entire field of view uniformly^33^. Moreover, the broadening also leads to intensity overlap of neighboring columns (Supplementary Fig. 7), which explains that fitting of Eq. 3 is truncated at larger *r* (“Methods”, Supplementary Fig. 7). The edge blurring differs from findings in previous 3D atomic-resolution imaging studies^2–8^ and must clearly be addressed to enable a stoichiometric determination in 3D atomic-resolution electron microscopy in general.

As atom vibrations occur on a timescale considerably faster than the second-long image acquisitions, their contrast fingerprint will be encoded in the phase reduction and image blur (Fig. 1a). Reduction of phase intensity has previously been attributed to electron-beam-induced phonon excitation of the object that temporarily stabilize atom excursions exceeding Debye–Waller factors during data acquisitions^10,34,35^. Such a thermalization of phonon excitations may also account for the present image blur. The atom displacements appear as a collective phenomenon with a marked site-dependency affecting the edge regions most dramatically as the enhanced *R*_av_ values span the three or four outermost atomic rows. Indeed, excitation of atom displacements is expected to be more pronounced at edges due to the reduced atom coordination compared to the bulk^14^. To make *R*_*v*_ directly quantifiable calls for phase contrast microscopes of extraordinary stability and resolution capable of tackling the significant data scatter in *V*′ and *R*_av_ (Supplementary Fig. 5 and 6).

The ability to take into account locally varying excitation strengthens a stoichiometric interpretation of the atomic columns even in the presence of the vibrational column blur. The enhanced atom vibrations (*R*_*v*_) around the equilibrium sites (Fig. 1a) can be corrected for by taking advantage of the radially inverse relationship of *R*_*av*_ and *V*′. The stoichiometrically relevant scattering potentials *V* are suitably deduced by multiplying *V*′ by π*R*_av_^2^  and reveal a uniform contrast pattern consistent with the dumbbell motif of 1Mo-2S columns across the bulk part of the MoS_2_(001) basal plane (Fig. 1f).

**Fig. 2:**
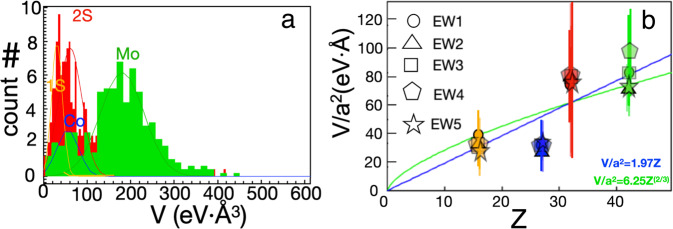
Stoichiometric interpretation of the Co-Mo-S exit wave. **a** Histograms of *V* values obtained from the metal and sulfur sub-lattice, marked by green and red, respectively, in the *V* map (Fig. 1f). The histogram peaks are assigned to 1Mo, 1Co, 2S and 1S, respectively. The histogram peaks are fitted by Gaussian functions (superimposed) having peak positions and standard deviations as listed in Supplementary Table 1. **b** The projected potential (*V*) scaled by the atomic area (*a*^2^) is plotted versus the assigned projected atomic number of the column (*Z*). The *V*/*a*^2^ values are obtained from the peak positions and standard deviations of the Gaussian fits to the *V* histograms for each exit wave (Supplementary Table 1) and from *﻿a* being 145, 135, and 110 pm for Mo, Co, and S, respectively. The data is best fitted by *V*/*a*^2^ = α_1_*Z* with α_1_ = 1.97 ± 0.12 eV Å (blue line) and *V*/*a*^2^ = α_2_*Z*^2/3^ with α_2_ = 6.25 ± 0.15 eV Å (green line), respectively. Moreover, the *Z*^2/3^-dependency corresponds to the potential *V* = α_3_*Z*^2/3^ with α_3_ = *a*^2^α_2_ = 13.13 ± 0.32 eV Å^3^, obtained using *a* = 145 pm.

The detailed stoichiometric interpretation of the *V* map for all exit waves relies on Eq. 2 and the corresponding phase histograms that are converted by α = 1.97 ± 0.12 eV Å (Fig. 2a and Supplementary Fig. 8). The histograms reveal four distinct distributions, two of which are associated with the metal and sulfur sub-lattices, respectively. The distributions are therefore attributed to 1S, 2S, 1Mo and 1Co atomic columns. This interpretation is confirmed by the linear *Z*-dependence of the average *V* value divided by the square of the atom radius, e.g. *V*/*a*^2^ (Fig. 2b), and rationalized by a simulation of the exit wave from a single-layer MoS_2_ nanocrystal including element-specific DW factors of 30 Å^2^ for Mo and 60 Å^2^ for S, respectively (Supplementary Fig. 9). In fact, the linear *Z*-dependence of *V*/*a*^2^ is also consistent with our previous static interpretation that the phase value *θ* (in rad) associated with the intensity distribution scales with the mass rule *θ* = 0.002 *M*, where *M* (= 2*Z*) is the mass of an atomic column^7^. In the past, however, a *Z*^2/3^-dependence of the phase values was demonstrated, relying solely on the maximum of the atomic column intensity distribution^36^. Likewise, fitting of the average *V*/*a*^2^ with a *Z*^2/3^-dependence in Fig. 2b predicts a potential of 13.13 eV Å^3^, consistent with the Rydberg energy 13.61 eV for hydrogen atoms (*Z* = 1). Thus, a stronger localization of the exit wave close to atomic nuclei causes the established non-linearity of the scattering potential with *Z* but remains insufficient to characterize the element dependence of phase-contrast measurements for nano-scale objects with spatially varying vibrational blur. Moreover, the standard deviation of the *V* distributions is about 1/4 of the distance between the 1Mo and 2S peaks, corresponding to *Z* = 42 and 32, respectively (Fig. 2a and Supplementary Table 1). Thus, the present approach achieves an element differentiation of *δZ* = 1/4 * (42–32) = 2.5 at 50 keV.

Clearly the *V* distributions of 1Mo and one Co (1Co) atomic columns in the metal sub-lattice and of 2S and one S (1S) atomic columns in the S sub-lattice overlap and reduce the accuracy of the stochiometric assignments (Fig. 2a and Supplementary Fig. 8). The distributions have peak positions and standard deviations (σ) as listed in Supplementary Table 1. For all exit waves, the peaks associated with 1Mo, 1Co, and 2S are distinguishable. The peak-to-peak separations of 1Mo-2S (*x*_0,1Mo_ − *x*_0,2S_) and 1Mo–1Co (*x*_0,1Mo_ − *x*_0,1Co_) are greater than the sum of their corresponding standard deviations *σ*_*1Mo*_, *σ*_*1Co*_, *σ*_*2S*_, and *σ*_*1S*_. For instance, for exit wave EW1, the separations of 1Mo–1Co and 1Mo–2S are 120.2 eV Å^3^ and 114.24 eV Å^3^, while the (*σ*_1Mo_ + *σ*_1Co_) = 71.95 eV Å^3^, and (*σ*_1Mo_ + *σ*_2S_) = 73.32 eV Å^3^, respectively. In contrast, for all exit waves, the peak-to-peak separation of 2S–1S (*x*_0,2S_ − *x*_0,1S_) is smaller than the sum of their corresponding standard deviations σ_2S_ and σ_1S_. As an example, for EW1, the separation of 2S–1S is 28.98 eV Å^3^, and (*σ*_1S_ + *σ*_2S_) = 37.51 eV Å^3^, respectively. Thus, the intersection of the Gaussian functions fitted to the *V* histograms must be considered in order to facilitate the stoichiometric assignment of the atomic columns. For the metal sub-lattice, columns are assigned to 1Mo or 1Co for *V* values greater or smaller than the intersection of Mo and Co Gaussian functions. Likewise, the same assignment is applied for the 2S and 1S columns in the S sub-lattice. For the metal sub-lattice, the Mo and Co Gaussian peaks are well-separated (Supplementary Fig. 8) and complementary electron energy loss spectroscopy studies have assigned Co atoms uniquely to sites at the S-edge^27^. For the sulfur sub-lattice, the overlap of the Gaussian peaks is more pronounced. Thus, the present stoichiometric assignment of the metal sub-lattice has a high confidence as compared to the assignment in the S sub-lattice under the present image acquisition conditions. The accuracy of the stoichiometric assignment to the metal sites (Mo and Co) and S sites is characterized by *δ*, which is defined as the change in the number of Co or 1S atoms (*Δn*) upon shifting the peak value *V* associated with Co or 1S by a small amount ± *ΔV*, i.e. *δ* = *Δn*/*ΔV*. The variation in the peak position of Co or 1S in the histogram is equivalent to the shift of the intersection of the Mo and 2S Gaussians. In general, an error of *ΔV* = 10 eV Å^3^ can give rise to the variation of one Co atom at the metal edge sites, corresponding to *δ* ~ 0.1 eV^−1^ Å^−3^ for assignment of Co to the edge in the metal sites. For the S sub-lattice, the assignment of the 1S and 2S is expected to sensitively depend on the peak positions and the standard deviations, as the sum of standard deviation exceeds the separation of 2S and 1S Gaussian peaks. In general, an error of *ΔV* = 1 eV Å^3^ can cause a variation of 1 to 5S vacancies (1S atomic columns) corresponding to *δ* ~ 1–5 eV^−1^ Å^−3^. Moreover, distributions of measurements in e.g. Supplementary Fig. 3, 4, and 8 contain a minority of outlier values that do not affect the overall conclusions of the present analysis and hence are not analyzed further herein.

**Fig. 3:**
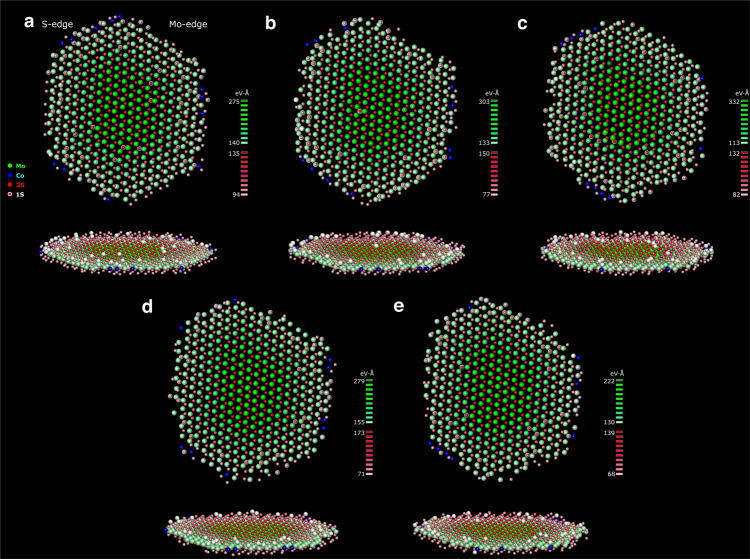
3D atom-dynamic maps of the Co-Mo-S nanocrystal. **a**–**e** The time-series of reconstructed 3D atomic-resolution images generated for the successive exit waves shown in the Supplementary Fig. 2. The 3D images depict the atoms with a color reflecting the stoichiometry of each atomic column and a color modulation in accordance with the *V’* values (Fig. 1d). Specifically, 1Mo is denoted by green dots, 1Co by blue dots, 2S columns by red dots, 1S columns by red dots over white dots and S vacancies by white dots. The corresponding exit waves are obtained from image acquisitions commencing at 153 s, 378 s, 423 s, 574 s, and 624 s, relative to time *t* = 0 s when the electron beam was switched on (Supplementary Fig. 2).

Based on this type of analysis, 3D atomic-resolution images of the nanocrystal can be generated from the successive exit waves (Fig. 3). Specifically, each 3D image represents the atomic columns at their 3D locations with a color reflecting the element content and a color modulation reflecting the image blur (*V’*). The corresponding time-resolution of the successive 3D images is on the scale of 10–100 s, as the exit waves are obtained from image acquisitions commencing at 153 s, 378 s, 423 s, 574 s, and 624 s, relative to time *t* = 0 s when the electron beam was switched on (Supplementary Fig. 2). Moreover, Fig. 3 shows successive 3D atomic-resolved images of the same MoS_2_ nanocrystal that remains flat (Fig. 1c, Supplementary Fig. 3) and rotates and tilts by less than ca. 1^o^ during the recording time, which is insufficient to affect the analysis (Supplementary Fig. 10). Thus, the successive 3D atomic-resolution images become a dynamic representation of atoms, reflecting the vibrational heterogeneity within each image and atom displacements among successive images.

### Resolving atom-dynamic heterogeneity across the Co-Mo-S nanocrystal in 3D

**Fig. 4:**
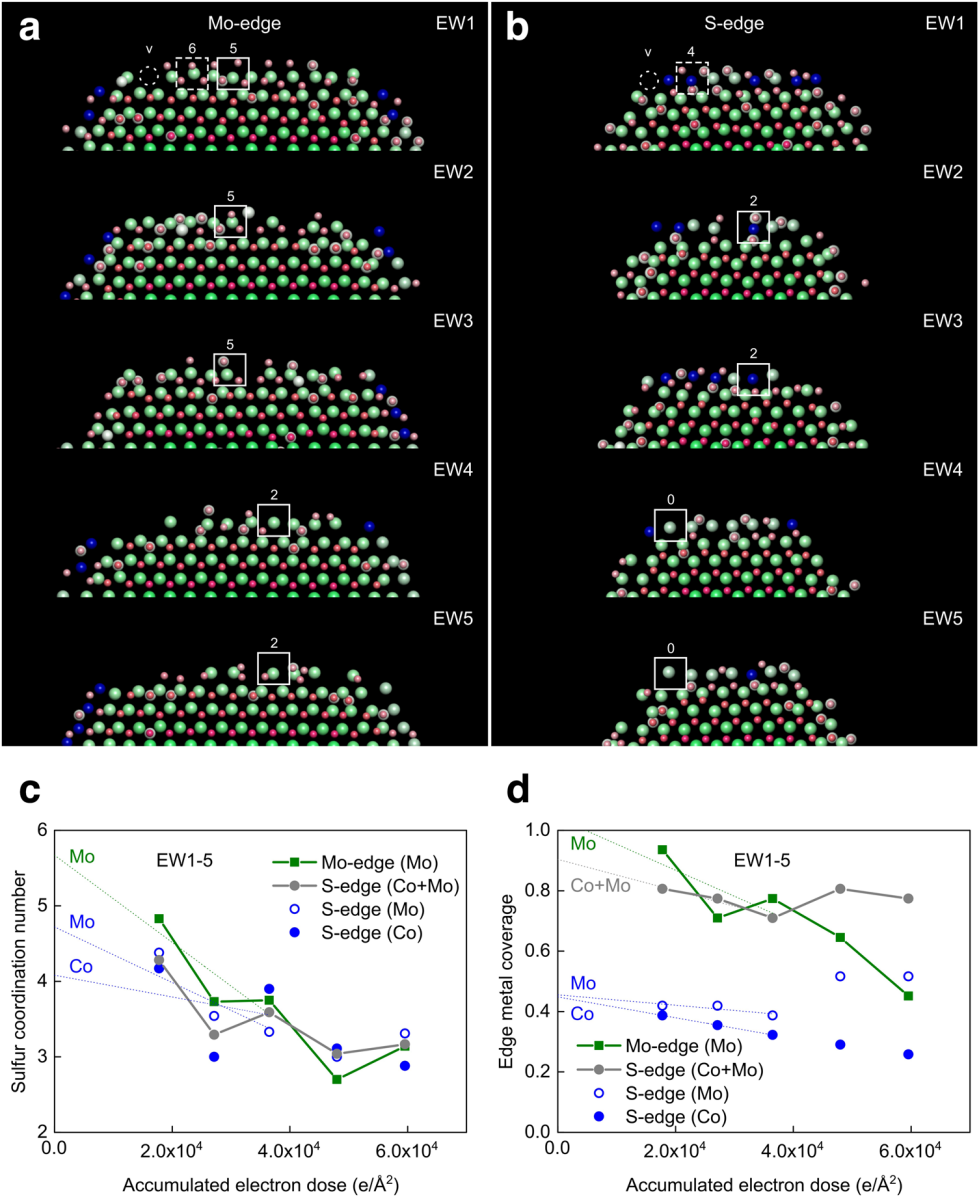
Temporal edge motifs on the single-layer Co-Mo-S nanocrystal. **a**–**b** Close-ups of Mo- and S-edges of the 3D atom-dynamic images in Fig. 3. Each edge site is characterized by the metal atom and its number of coordinating sulfur atoms (Supplementary Fig. 11). Examples are highlighted by the superimposed boxes (solid lines: motifs with S-coordination that remain in successive 3D images, dashed lines: motifs with S-coordination similar to equilibrium structures predicted by density functional theory calculations^26,27,37–39^.). The v denotes a metal vacancy. **c** Average S coordination number for the Mo atoms at the Mo-edge and Mo and Co atoms at the S-edge shown versus electron dose. **d** The fraction of metal atoms at the Mo- and S-edge, respectively, versus electron dose. The atomic fraction is normalized to the number of metal atoms at the Mo-edges and S-edges, respectively, of a fully edge-decorated Co–Mo–S nanocrystal (Supplementary Fig. 11). Superimposed lines (dotted) indicate best linear fit to the data obtained from the low dose rate images (EW1-3) in (**a**) and (**b**). Moreover, the electron dose is indicated for the center frame in the corresponding focus series.

The 3D atomic-resolution images reveal that the MoS_2_ nanocrystal is hexagonally shaped and terminated by (100) Mo- and (−100) S-edges with promoting Co atoms exclusively attached to the S-edges, consistent with Ref. ^27^. With increasing illumination time, the edges expose a multitude of different motifs with short-range order and the nanocrystals exhibit a slight atom loss (Figs. 3, 4 and Supplementary Movie 1 and Table 3). Specifically, the edge motifs include metal atoms with a four- and six-fold coordination by S, similar to equilibrium structures^26,27^, and metal atoms with S deficiency, representing meta-stable atom arrangements (Fig. 4a, b and Supplementary Fig. 11). In fact, stability of edge motifs in successive images is only detected for ~12% of the edge sites, including two-fold coordinated Co atoms at the S-edge and five-fold coordinated Mo atoms at the Mo-edge (Fig. 4). Edge restructuring is accompanied by a linear decrease in the S coordination number as well as in the metal occupancies for the lower dose rate data (EW1-3; Fig. 4c, d). This progression enables an extrapolation to zero dose based on linear fitting. Hereby, the pristine edges are associated with a S coordination number of approximately 4-5 and 5 for Co and Mo atoms at the S-edges, respectively, and 5-6 for Mo atoms at the Mo-edge (Fig. 4c). Moreover, the pristine edges contain a fully Mo-occupied Mo-edge and a roughly 50:50 Co:Mo alloy at the S-edge (Fig. 4d). These findings agree with predicted equilibrium structures of the Co-Mo-S and MoS_2_ phases under the present sulfidation and imaging conditions^26,27,37–39^. Thus, the nanocrystal edges structurally degrade under electron illumination. However, capturing the time dependence enables a single-atom extrapolation to the pristine atomic structure, which works best if the lower dose rate is employed. At the higher dose rate, for example, the S coordination number remains constant and the metal coverage continues decreasing at the Mo-edge, while it remains almost constant at the S-edge (Fig. 4c, d). At the S-edge, Co atom removal is in fact accompanied by an incorporation of Mo atoms into vacant sites, which changes the one-dimensional edge alloy composition (Fig. 4d). Therefore, a critical assessment of the illumination conditions is needed to establish proper extrapolation schemes for retrieving pristine 3D atomic structure.

The analytical method presented here measures locally varying excitation strengths and enables an account of atom dynamics induced by the electron beam in the present case. Moreover, the edge alterations are accompanied by the formation and redistribution of S vacancies (Fig. 3 and Supplementary Fig. 12). These are primarily present on one side of the MoS_2_ nanocrystal reflecting the exposed basal plane. Moreover, the S vacancies are mainly located at the atomic rows with the largest vibrational blur (Supplementary Fig. 12). This finding indicates that the edge-confined vibrations lead to bond-breaking events below the knock-on threshold values for atom displacements from bulk sites (“Methods”), and thus govern the dynamic progression of Co–Mo–S edges. This ability to relate local excitations and evolutions of atomic motifs is readily applicable and can in the future be beneficially used in phase contrast electron microscopy operating at the limits of electron optics and detection. The analytical model is therefore anticipated to open up possibilities for probing dynamic heterogeneities in nano-scale materials at the single-atom level both inherent and induced by chemically meaningful stimuli to advance the fundamental understanding of the structure-sensitive reactivity in clusters, surfaces, and molecules in general.

## Methods

### Sample preparation

An industrial-style Co-promoted MoS_2_ (Co–Mo–S) hydrotreating catalyst was prepared by incipient wetness impregnation of a high-surface-area graphitic support with (NH_4_)_2_[MoS_4_] precursor and Co(acetate)_2_ followed by sulfidation as in Ref. ^26^. The sulfidation led to the formation of MoS_2_ structures, including single- and few-layer nanocrystals. After sulfidation the catalyst was exposed to dry N_2_ for storage, sample preparation, and transportation. The sample was only exposed to ambient for a few minutes for insertion into an electron microscope.

### TEM imaging and exit wave reconstruction

Co–Mo–S nanocrystals supported on graphite were examined using the TEAM 0.5 microscope at Berkeley National Laboratory^40^. The microscope was operated in the transmission electron microscopy (TEM) mode. The primary electron energy was 50 keV, which is below the threshold energy for S atom knock-on displacement from the basal plane of above 60 keV^26,28–30^ and, hence, suppresses atom sputtering of single S and Mo atoms^28^. Nelsonian illumination sets the electron dose rates at ca. 94 and 290 e^−^Å^−2^ s^−1^. These dose rates correspond to an average electron impingement rate of 5 × 10^6^ e^−^ s^−1^ and 17 × 10^6^ e^−^ s^−1^, respectively, over the illuminated sample area that was slightly larger than the image area (approximately 24 × 24 nm^2^). Moreover, prior to image acquisition, the microscope was corrected for electron optical aberrations to 5th order with the spherical aberration coefficient set to ca. −11 µm with an isotropic information transfer of 0.34 Å^−1^. Under these conditions, the microscope information limit is ca. 1.4 Å^41^, which is sufficient to resolve the 1.8 Å separation of 1Mo–2S dumbbell. The high stability of the TEAM 0.5 microscope allowed for acquisition of image series at well-defined optical settings^41^. Specifically, five successive focal series of images were acquired in focal steps of −5.5 Å (94 e^−^Å^−2^ s^−1^) and −11 Å (290 e^-^Å^−2^ s^−1^) and with defocus starting at approximately 350 Å using a Gatan US1000 charge-coupled device (CCD) camera with a pixel size of 0.230 Å and exposure time of 1 s. In order to avoid any interference of the graphite support lattice, the corresponding structure factors were removed by annular band-pass filtering of all images in the focal series of spatial frequencies between 0.44 and 0.50 Å^−1^ and > 0.77 Å^−1^ (Supplementary Fig. 1). The MoS_2_ structure factors were not affected by the filtering. The modulation transfer function of the CCD pixel detector was measured by the edge method with an aperture^42^ and corrected for by Fourier deconvolution. Subsequently, each series of images were coarsely aligned using cross-correlation to compensate for lateral drift and each series was then processed to reduce residual drift, determine the image focal values and reconstruct the exit wave using the Gerchberg–Saxton algorithm as implemented in the exit wave reconstruction package of the MacTempas software (www.totalresolutions.com)^43^ (Supplementary Fig. 1, 2).

### Exit wave analysis

To quantify the atomic column heights, the complex exit waves were analyzed following Ref. ^44^. The positions of the atomic columns in the basal plane of the <001>-oriented Co-Mo-S nanocrystal were determined using the peak-finding function in the MacTempas software followed by a refinement of the positions with sub-pixel precision by fitting a Gaussian function to each peak in the imaginary image. For each atomic column in an exit wave, the complex value was determined as the average of a peak-centered 3 × 3 pixels^2^ area at a pixel sampling of 0.0624 Å/pixel, which is equivalent to 3.5 × 10^−2^ Å^2^. Moreover, the valley sites in the MoS_2_ structure in the exit waves were taken as vacuum sites and their values were also obtained as the average of a peak-centered 3 × 3 pixels^2^ area at each vacant site of the MoS_2_. For each column, the exit wave value was propagated by ± 50 Å around Gaussian focus in 0.1 Å steps by multiplication with the wave propagator exp(iπ*εk*^2^) in Fourier space (*ε*: defocus, *k* = |***k*** | : spatial frequency). At each focus step, the exit wave modulus was averaged from a 3 × 3 pixels^2^ area at all column positions. The resulting plot of modulus versus defocus was fitted by a second-order polynomial function yielding a maximum that represents the atomic column heights relative to a common image plane^44^. The atomic column heights were then refined using the Big-Bang scheme^5^. Supplementary Fig. 3 shows histograms of atomic column heights. Subsequently, the exit wave values for all the atomic columns were corrected for their defocus and thus referenced to a common image plane. The focus-corrected exit wave values are presented in an Argand plot and hence properly reflect the atomic content (Supplementary Fig. 4). Specifically, this plot is expected for a thin specimen for which the electron wave function is approximated by Ψ(**r**) = 1+iV(**r**), in which the phase and imaginary part are given as V(**r**). Figure 1 and Supplementary Fig. 2 show phase images of the exit wave and following Eqs. (1)-(3), the imaginary part is used for mass quantification.

A quantification of the stoichiometry and radius of the atomic columns is based on the analytical model Eqs. 1–3. According to Supplementary Eq. 17, the projected atomic number *Z* is linearly related to the projected electrostatic potential *V* associated with each atomic column. Moreover, each column is associated with a radius *R* reflecting the electron scattering potential, atom vibrations and residual lens aberrations. These structural parameters are related as: Im(<Ψ_N_(**r**)>) = *V*/(π*R*^2^) exp(−*r*^2^/*R*^2^) with <Ψ_N_(**r**)> as the exit wave normalized with respect to the vacuum wave defined in Supplementary Eq. 11 and 15. The Gaussian model for the imaginary part of the exit wave peaks at a value *V’* = *V*/(π*R*^2^), in units of eV Å, in the case of the isotropically broadened atomic columns (Eq. 3 and Supplementary Eq. 18). The V’ value can be determined by inspecting the natural logarithm of Im(<Ψ_N_(**r**)>) projected onto the r^2^ axis. That is, ln{Im(<Ψ_N_(**r**)>)}, is expected to be linearly related to *r*^2^. From several Mo and S atomic sites (Supplementary Fig. 8a), typical plots of ln{Im(<Ψ_N_(**r**)>)} vs *r*^2^ are shown in Supplementary Fig. 7b. In each plot, the data are extracted from an atomic column within a square area of 21 × 21 pixel^2^ (about 1.3 Å x 1.3 Å) divided into 71 circular zones in radial direction with an equal *r*^2^ up to near 1Å^2^. The pixel sampling is 0.0624 Å/pixel, and 1 Å corresponds to the length from the peak center of the atomic column to the corner in diagonal direction. Linear regression provides the slope and intercept, corresponding to 1/*R*^2^ and ln(*V’*), respectively, for the Gaussian model of an azimuthally symmetric atomic column. In practice, however, the atomic column shapes are associated with an azimuthally varying radius *R* as reflected by the non-linear fans in Supplementary Fig. 7b. The anisotropic shape (Supplementary Fig. 6b) is also reflected by the insets of Supplementary Fig. 7b that are binary images displaying only the pixels with a value within 75–83% of the peak value *V’* of the atomic columns in the imaginary image. For a perfect Gaussian function, the width at 80% of the peak value is equivalent to a size of about 0.95 *R*. Moreover, the 21 × 21 pixel^2^ area may also include contrast contributions from neighboring atomic columns. That is, the anisotropic shapes evolve from temporary residual aberrations, atom vibrations, as well as overlap with its nearest neighboring atomic columns. In fact, the overlap leads to an azimuthally varying radius that can span negative as well as positive slopes (Supplementary Fig. 7c). In view of these findings, the peak values *V’* are determined by restricting the linear regression to about *r*^2^ = 0.2 Å^2^ (*r* = 0.45 Å) to remove overlapping atomic columns. In fact, all fans in Supplementary Fig. 7b have a common intercept, which provides a precise determination of the peak value *V’*. The *V’* values are presented at the corresponding atomic column positions as maps (Fig. 1d) and azimuthally averaged and plotted versus the radial distance from the edge toward the center of the nanocrystal (Supplementary Fig. 5). Furthermore, *R* is given as an averaged atomic column radius *R*_*av*_, which is determined as the average of *R*_max_ and *R*_min_, being the longest and shortest radius of an anisotropic atomic column in binary images (Supplementary Fig. 6a, b), respectively. That is,

5 $${R}_{{{{{{\rm{av}}}}}}}=({R}_{{{{{{\rm{max}}}}}}}+{R}_{{{{{{\rm{min}}}}}}})/2.$$

Thus, multiplication of *V’* (=*V*/(π*R*^2^)) and (*πR*_*av*_)^2^ yields the estimated integrated potential value *V* for every atomic column. The integrated potential *V* is expected to be decently uniform across the MoS_2_ nanocrystal because of the opposing radial trends in *V’* (Supplementary Fig. 6) and *R*_*av*_ (Supplementary Fig. 6a, b), as also evidenced by the *V* map (Fig. 1f). Moreover, all Mo and S atomic columns are used to generate the histograms of distorted Mo-S bond angles (Supplementary Fig. 6c).

The *V* values associated with atomic columns in either the metal or S sub-lattices are divided into about 40–50 channels depending on the number of atomic columns, as shown in Fig. 2a and Supplementary Fig. 7b. That is, the number of Mo and S columns are about 317–305 and 355–318, respectively, for exit wave EW1 to EW5 and the number of columns divided by 7 is used as the number of channels in the *V* histograms. The number of channels in each histogram is thus around 45–43 for Mo and 50–45 for the S sub-lattices, respectively. The resulting histograms reveal several peaks that are fitted with a Gaussian function. The corresponding peak positions and standard deviations (*σ*) are listed in Supplementary Table 1.

## Supplementary information

Supplementary information

Description of Additional Supplementary Files

Supplementary Movie 1

## Data Availability

The data reported in this study are available from the authors upon reasonable request.

## Code availability

The analysis procedures are based on mathematical equations and image processing as described in “Methods”. The corresponding codes are written in the Tempas platform (www.totalresolution.com) and are available from the authors upon reasonable request.
