# Supplementary material for: Probing atom dynamics of excited Co-Mo-S nanocrystals in 3D
Source: Nat Commun. 2021 Aug 18;12:5007. doi: 10.1038/s41467-021-24857-4 (PMC8373969; doi:10.1038/s41467-021-24857-4)
Supplement: Supplementary file 1 — Supplementary information [file 41467_2021_24857_MOESM1_ESM.pdf]

Supplementary Information

Probing atom dynamics of excited Co-Mo-S nanocrystals in 3D

Fu-Rong Chen[1], Dirk van Dyck[2], Christian Kisielowski[3], Lars P. Hansen[4], Bastian Barton[3], Stig Helveg[4,5,*]

[1]Department of Materials Science and Engineering, Hong Kong City University, Hong Kong.

[2]EMAT, University of Antwerp, Groenenborgerlaan 171, B2020 Antwerp, Belgium.

[3]The Molecular Foundry, Lawrence Berkeley National Laboratory, One Cyclotron Road, Berkeley, CA 94720 (USA).

[4]Haldor Topsoe A/S, Haldor Topsøes Allé 1, DK-2800 Kgs. Lyngby, Denmark.

[5]Center for Visualizing Catalytic Processes (VISION), Department of Physics, Technical University of Denmark, DK-2800 Kgs. Lyngby, Denmark.

*Correspondence to: stig@fysik.dtu.dk.

Supplementary Figures

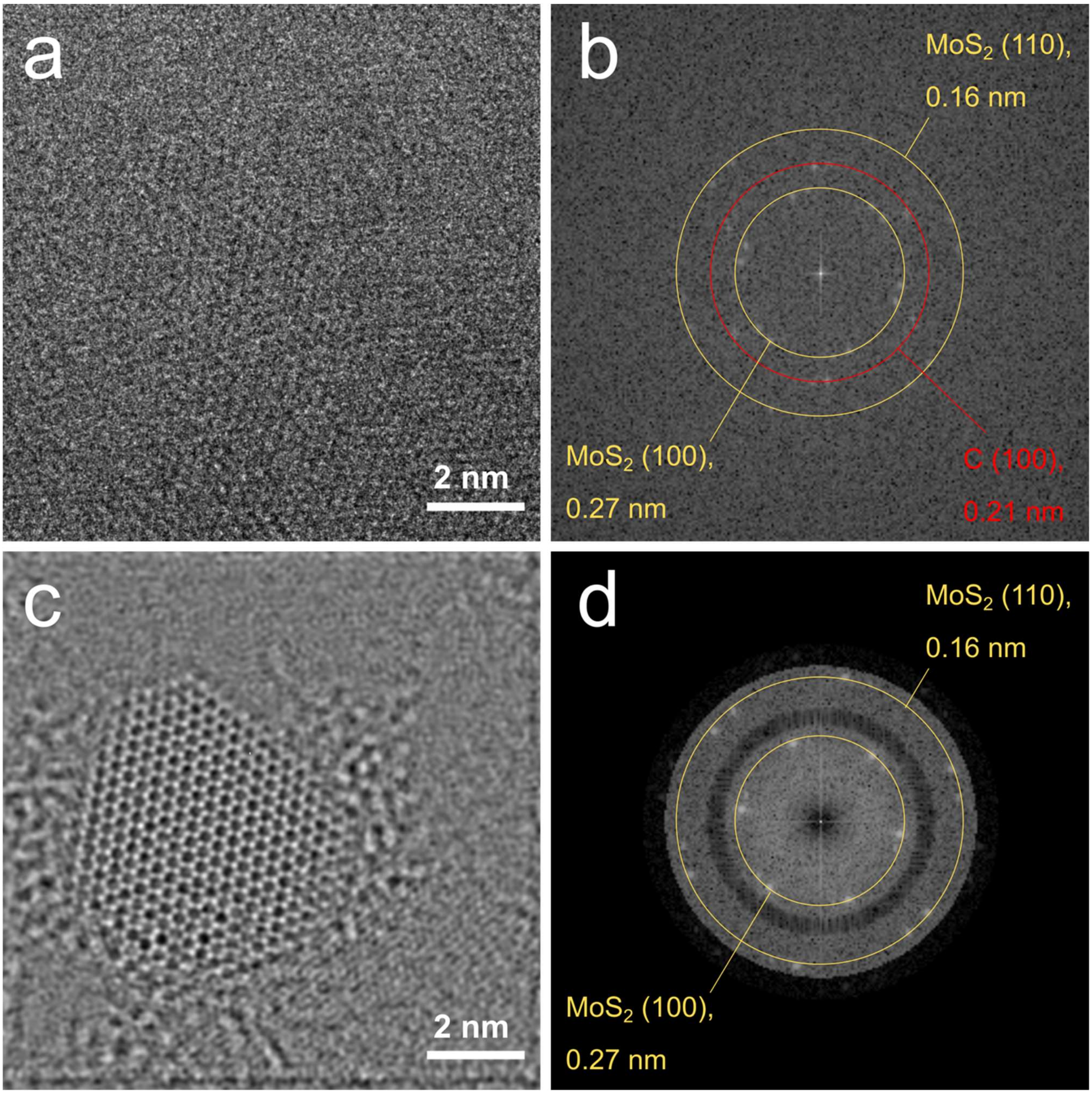

Supplementary Figure 1. Electron microscopy at the noise limit. a, Single image acquired at approximately + 4 nm overfocus from the low dose rate focal image series used to reconstruct exit wave EW2. **b,** Fast Fourier Transform (FFT) of (a) revealing crystalline spots from the MoS_2 nanocrystal and the graphite support. **c,** Phase image of the reconstructed exit wave EW2. **d,** FFT of (c) with graphite reflections filtered out of the focal image series prior to exit wave reconstruction.

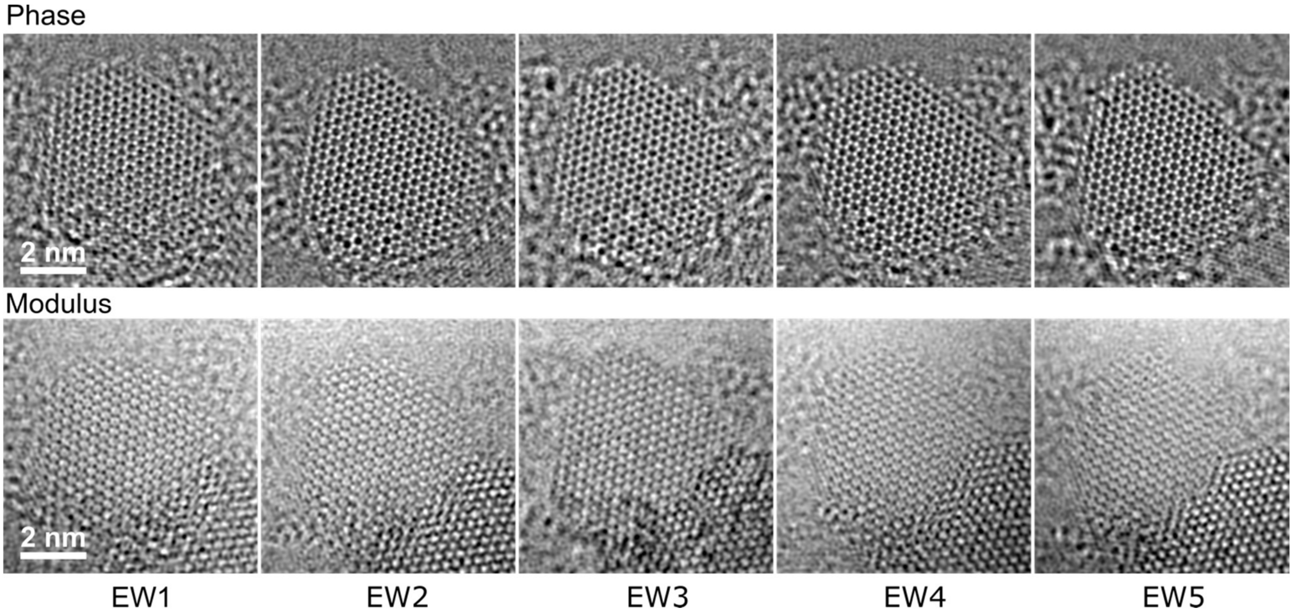

Supplementary Figure 2. Five successive exit waves of a <001>-oriented Co-Mo-S nanocrystal on graphite. The top and bottom rows show the phase and modulus images, respectively, of the reconstructed exit waves (EW1-5). The sample was continuously exposed to the electron beam which was switched on at time t = 0 s. The five corresponding focal series of images were acquired at time intervals t=153-193s (EW1), 378-422s (EW2), 423-463s (EW3), 574-594s (EW4), 624-646s (EW5), containing 40, 44, 40, 20 and 22 images, respectively. The image series were assigned start focus values of 298Å (EW1), 223Å (EW2), -3.5Å (EW3), 210Å (EW4) and 353Å (EW5) for exit wave reconstruction. Each focal series of images were acquired with an electron dose rate of 89, 94, 94, 287 and 290 $e^{-}Å^{-2}s^{-1}$, corresponding to a total dose of 3560, 4136, 3760, 5740 and 6380 $e^{-}Å^{-2}$, and an accumulated total dose of 17177, 37090, 41132, 60008, and 71880 $e^{-}Å^{-2}$, respectively. The exit-wave reconstructions were based on images cropped from the full recorded images that contained a specimen free region used to measure the actual electron dose rate.

Coincidentally, the Co-Mo-S nanocrystal overlaps in its lower right corner with another nanocrystal. Optical sectioning shows this additional nanocrystal has minimal modulus and hence is displaced out of focus by another 75 - 100 Å along the beam direction (data not included here). For the present analysis this overlap has negligible effect because: (i) Mass determination of the atomic columns uses the phase value at Δf=0Å of the nanocrystal of interest for which the phase is maximized and

reduced of the other nanocrystal (Fig. 1-2). (ii) For a thin specimen, as justified by Supplementary Fig. 4, the electron wave function is $\Psi=1+iV(r)$, where the phase and imaginary part is the same and given by V(r) and the square of the modulus is $(1+V(r)^2)$. The atomic column heights are determined by maximum intensity propagation of the entire exit wave minus 1, (Ψ-1), in which case only the phase (or imaginary part) V(r) dominates the intensity propagation. The propagation around Δf=0Å by ±50 Å only implies that the phase of the nanocrystal of interest remains close to maximum and of the other nanocrystal the phase is strongly reduced. As a result, the intensity maximum for Ψ-1 at the true z-height is close to 0Å within 10 Å (Supplementary Fig. 3). Thus, for the present thin samples, the phase value of the nanocrystal of interest dominates the height determination, which, in turn, explains the narrow width of the height histograms (Supplementary Fig. 3) and indicates that contributions from the overlapping nanocrystal is indeed negligible. Obviously, for thicker nanocrystals, the general approach should avoid overlapping nanocrystals. Moreover, it is noted that the overlapping nanocrystal is not static through exit waves EW1-EW5, whereas the nanocrystal of interest retains its orientation (Supplementary Fig. 10) and only loose a tiny fraction of the constituent atoms (Supplementary Table 3).

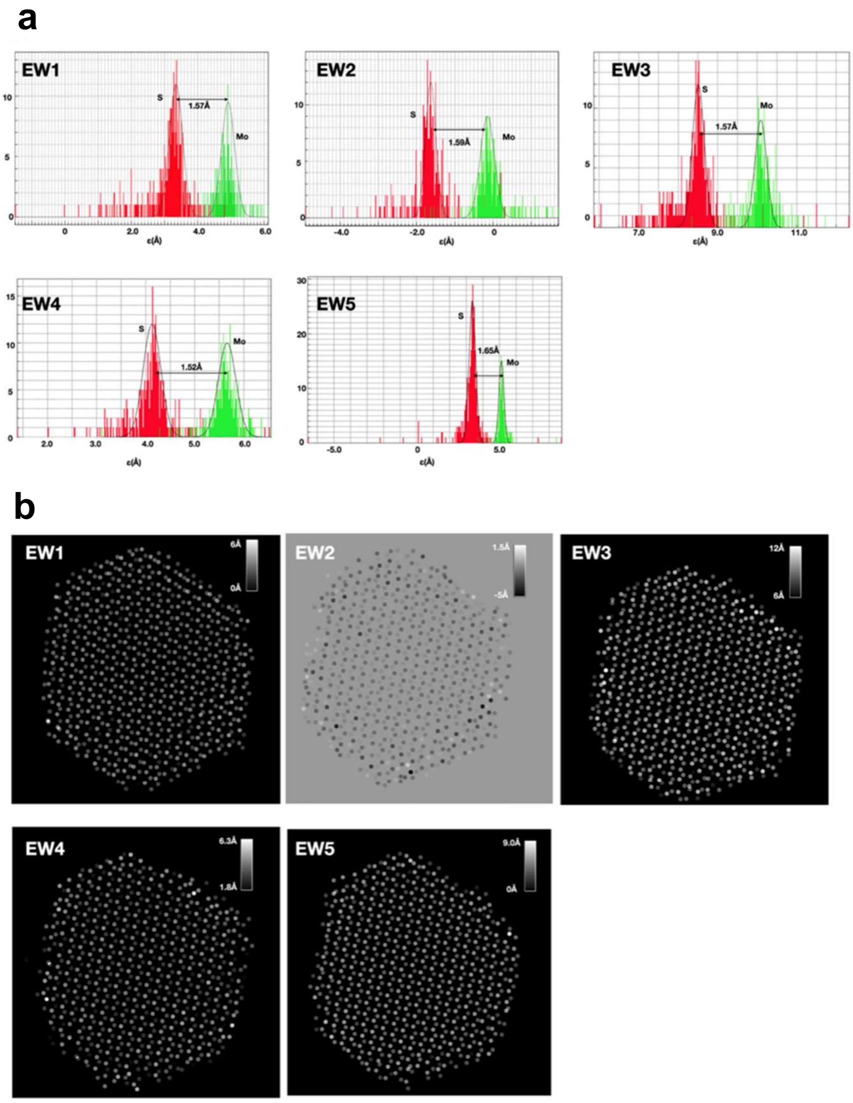

Supplementary Figure 3. Atomic column height histograms and maps. a, Atomic column height histograms for exit waves EW1-5 in Supplementary Fig. 2. The atomic columns are distributed in narrow peaks in both the Mo and S sub-lattices as denoted by green and red, respectively. The average peak-to-peak distance of 1.58 Å corresponds to the Mo-S basal plane distance in 2H-MoS_2. **b,** Atomic column height maps indicating that the atomic columns are positioned similarly across the basal plane within each sub-lattice.

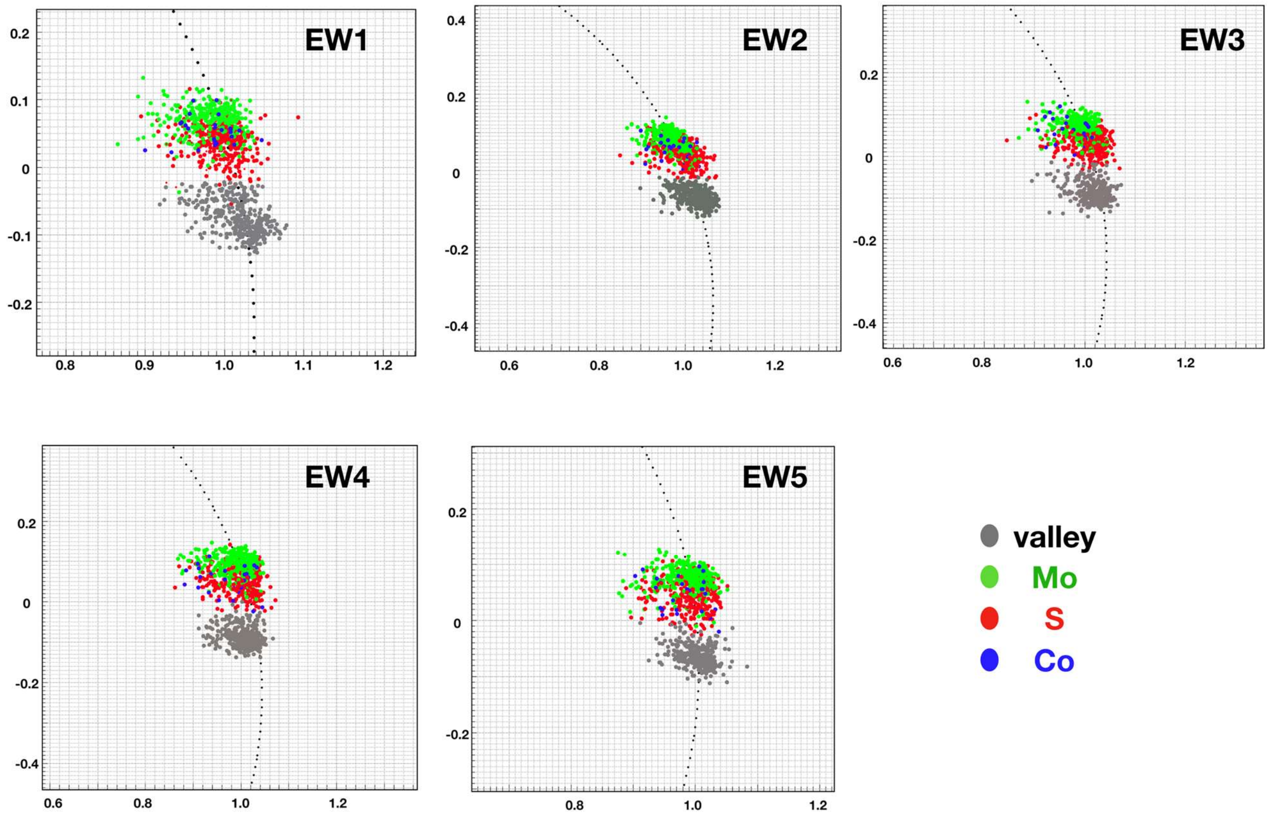

Supplementary Figure 4. Focus-corrected Argand plots. The complex value of the Mo, S, Co and valley (vacant, interstitial) columns in the exit wave is represented by grey, green, red and blue colored dots, respectively. A mass circle, shown as dash black lines, is fitted to all the green, red and grey dots for each exit wave. The fitted radius and center are given for EW1-5 in Supplementary Table 2.

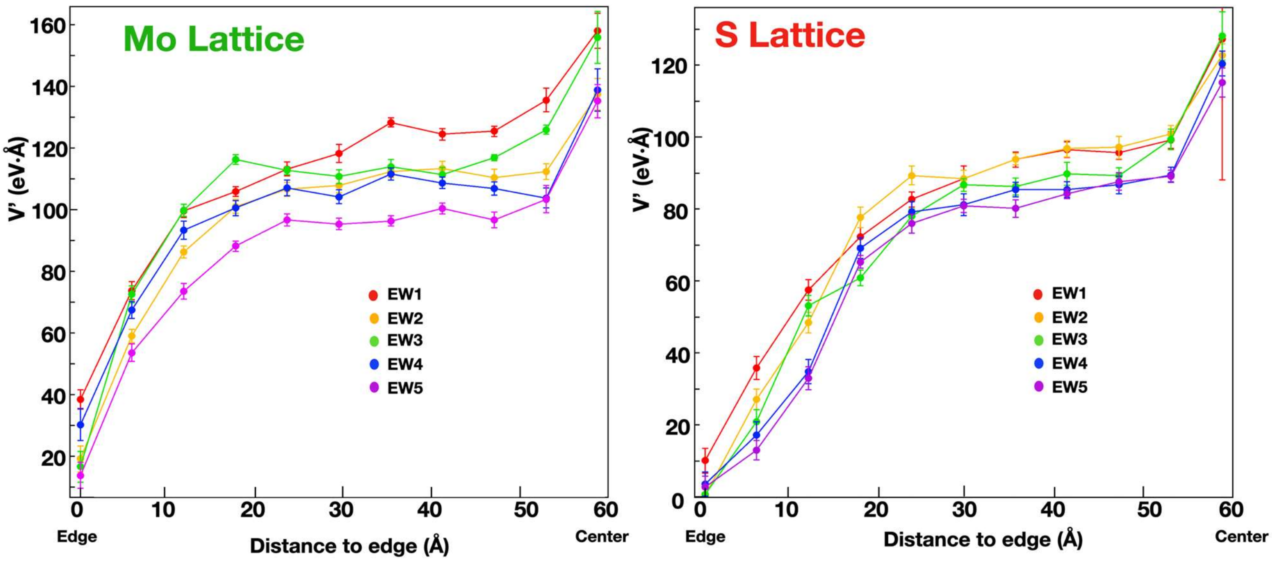

Supplementary Figure 5. The projected potential *V'* for the atomic columns of EW1-5. The *V'* potential for the atomic column in the Mo and S sub-lattices is azimuthally averaged from *V'* maps, such as Fig. 1d, and plotted versus the radial distance from the edge toward the center of the nanocrystal. The corresponding standard deviation is indicated by the error bars. A slight deviation from hexagonal shape implies that two 2S columns and two 1Mo columns are associated with the center site.

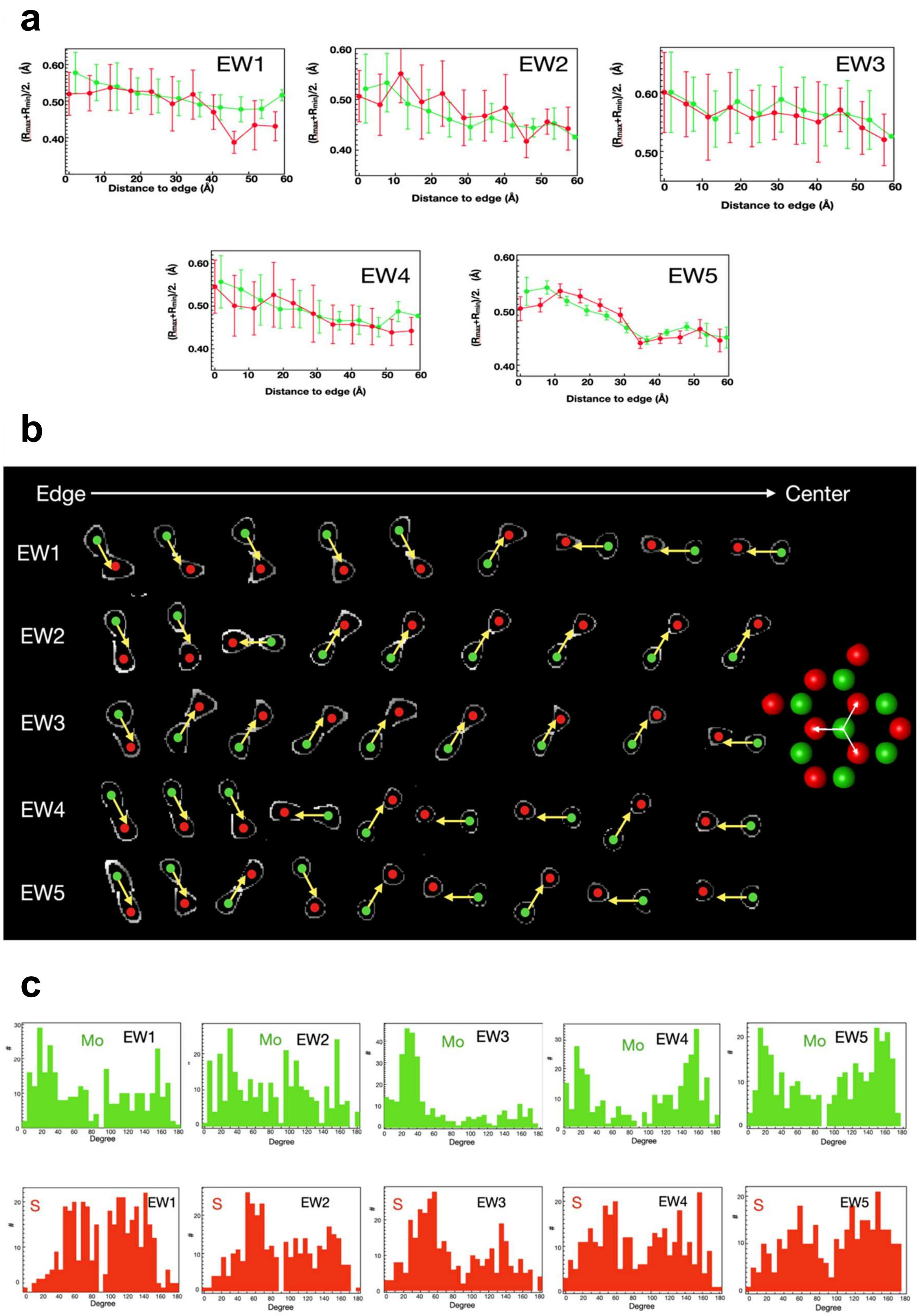
a
EW1
EW2
EW3
EW4
EW5
(R_max+R_min)/2. (Å)
Distance to edge (Å)
b
Edge
Center
EW1
EW2
EW3
EW4
EW5
c
Mo
S
EW1
EW2
EW3
EW4
EW5
Degree

Supplementary Figure 6. The average radius R_{av} of the atomic columns for EW1-5. a, For each exit wave, R_{av} of the atomic columns is derived from the longest and shortest axis in the binary image in the Mo and S sub-lattices. Fig. 1d shows an example of a R_{av} map (EW1). From the R_{av} map, the azimuthally averaged R_{av} value and its standard deviation are obtained for the Mo and S sub-lattices and plotted in green and red, respectively, as a function of distance from the edge toward the center of the nanocrystal. The R_{av} is larger near the edge and decays toward the center. **b,** A selection of Mo-S pairs from a S-edge toward the center for exit waves EW1-5 is displayed as binary images with pixel values within 75-83% of the peak value. Visual inspection reveals that most of Mo-S pairs elongates closely along the ideal bond direction of MoS_2. All yellow arrows originate at the Mo column (peak position given by green dot) and points at the S column (peak position given by red dot). Specifically, the white arrows in the MoS_2 structural model show an ideal 120° character of MoS_2. Comparing the yellow and white arrows reveals that the anisotropically shaped Mo-S pairs follow closely the ideal bond direction. **c,** Histograms of the elongation angle of all Mo and S atomic columns. The elongation angle of the Mo and S atom columns is defined as the angle of the longest axis of the anisotropic shape of the Mo and S atom column relative to the horizontal axis. The histograms peak at 30 and 140 degrees and near 60 and 120 degrees, respectively.

a

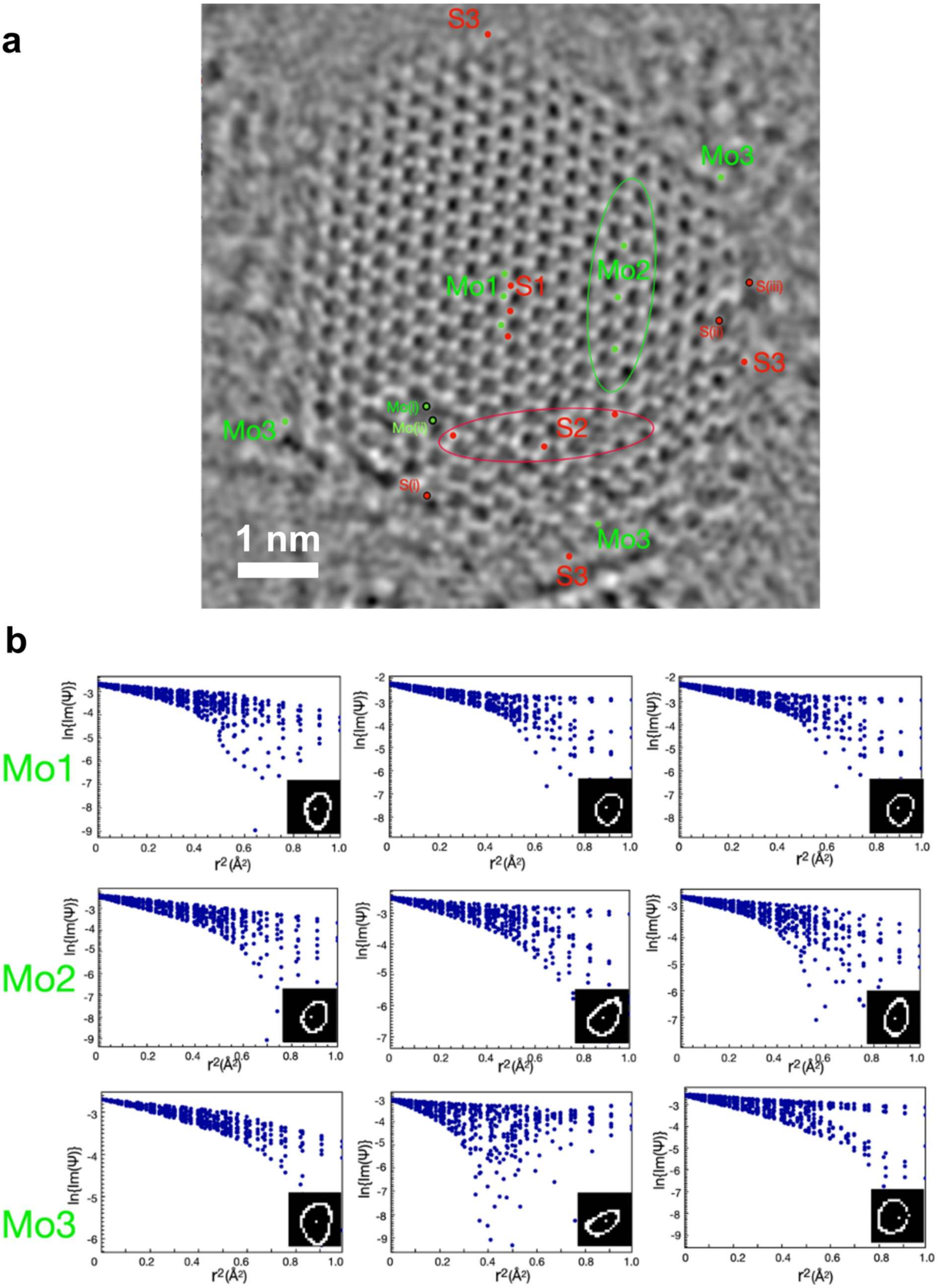
S3
Mo3
Mo2
Mo1
S1
S(iii)
S(ii)
S3
Mo(i)
Mo(ii)
Mo3
S2
S(i)
1 nm
Mo3
S3
b
Mo1
Mo2
Mo3
ln{Im(Ψ)}
r2(Å2)

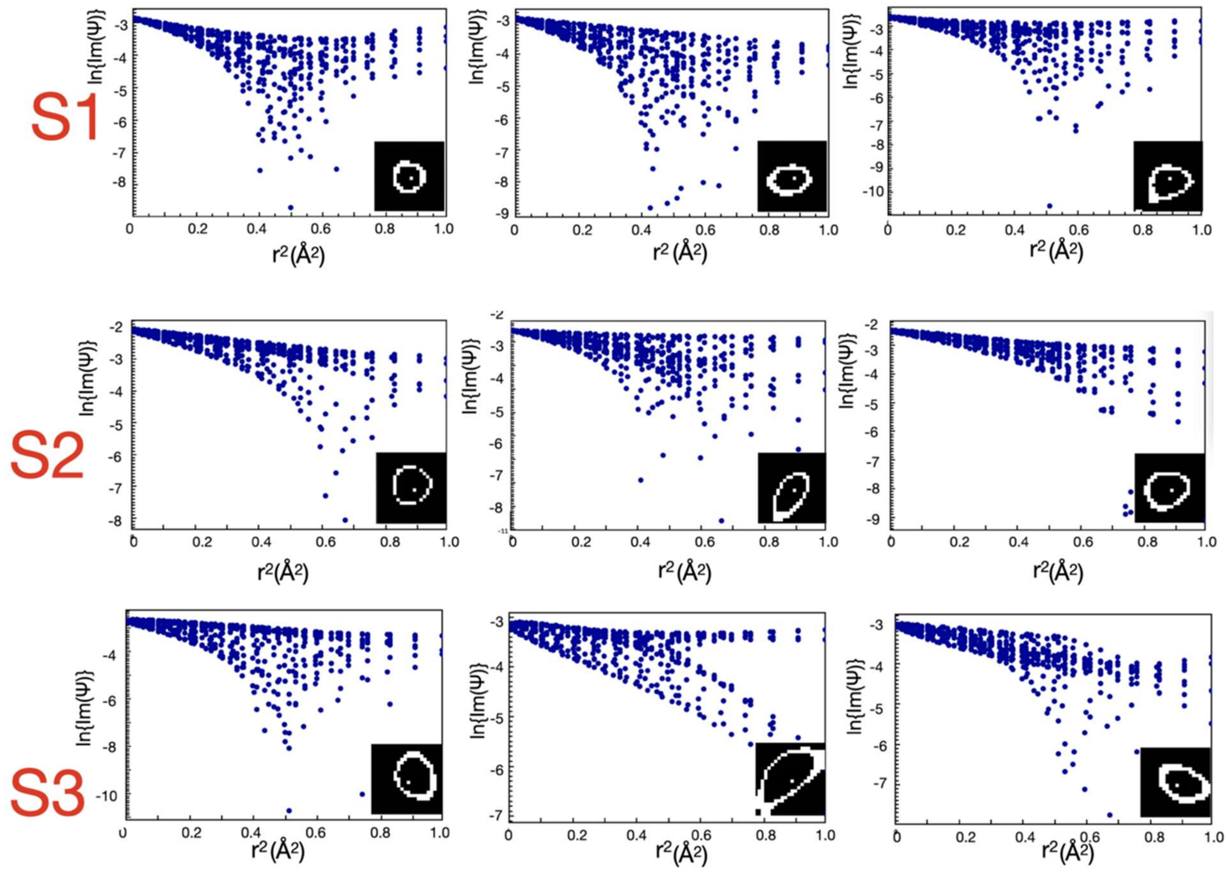

c

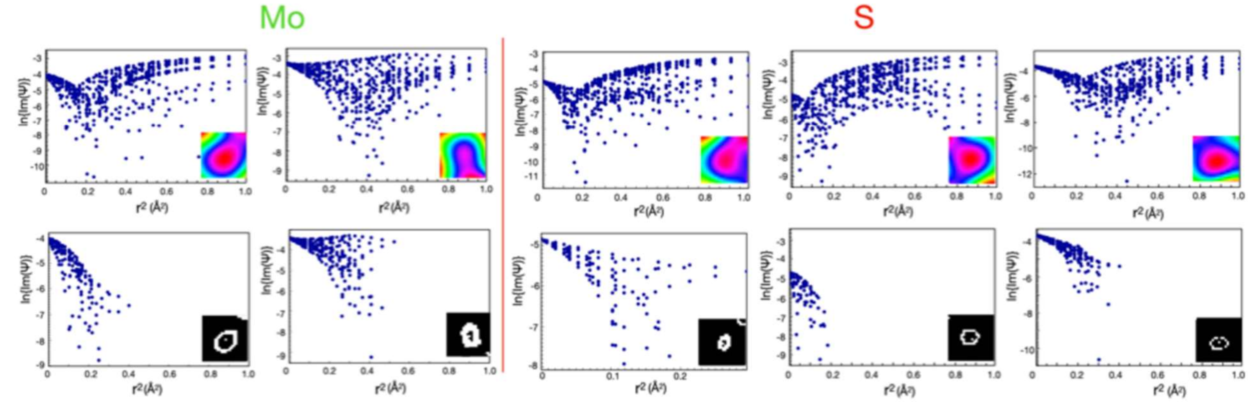

Supplementary Figure 7. Atom column shapes in EW1. a, Imaginary image of EW1 pinpoints a selection of Mo and S atomic columns, denoted by green and red dots, respectively, for the analysis in b, and a selection of Mo and S atomic columns, denoted by green and red dots encircled by black, respectively, for the analysis in c. **b,** Plots of $\ln\{\mathrm{Im}(<\Psi_N(\mathbf{r})>)\}$ vs r^2 for Mo and S columns in a. The insets display the binary image at 75-83% of the column peak value to show the anisotropic shape

of the atom column. The central dot represent the position of $\ln\{\mathrm{Im}(<\Psi_N(\mathbf{r})>)\}$ at $r = 0$. **c,** Plots of $\ln\{\mathrm{Im}(<\Psi_N(\mathbf{r})>)\}$ vs r^2 for Mo and S atom columns in a. The upper row shows the full dataset including marked overlap with neighboring atom columns. The overlap is highlighted by the inset showing a cropped imaginary image of the column. The lower row shows the dataset truncated at $r^2 > 0.2$ Å^2 to emphasize the data obtained from the single atom column.

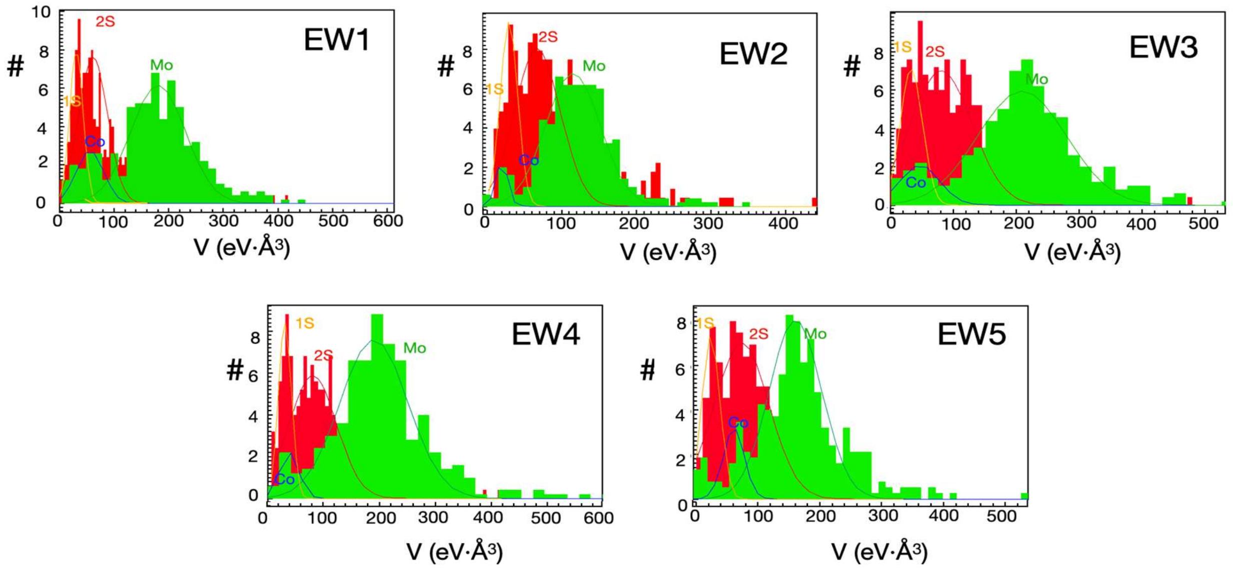

Supplementary Figure 8. Histograms of the integrated potential *V*. The V is determined by multiplying $V/(\pi R^2)$ by πR_{av}^2 for each individual atomic column in the metal (green) and S (red) sub-lattices of the MoS_2 nanocrystal. A Gaussian function is fitted to the identifiable peaks and the corresponding fitted peak positions, x_o, and standard deviations, σ, for the 1Mo, 1Co, 2S and 1S columns are listed in Supplementary Table 1.

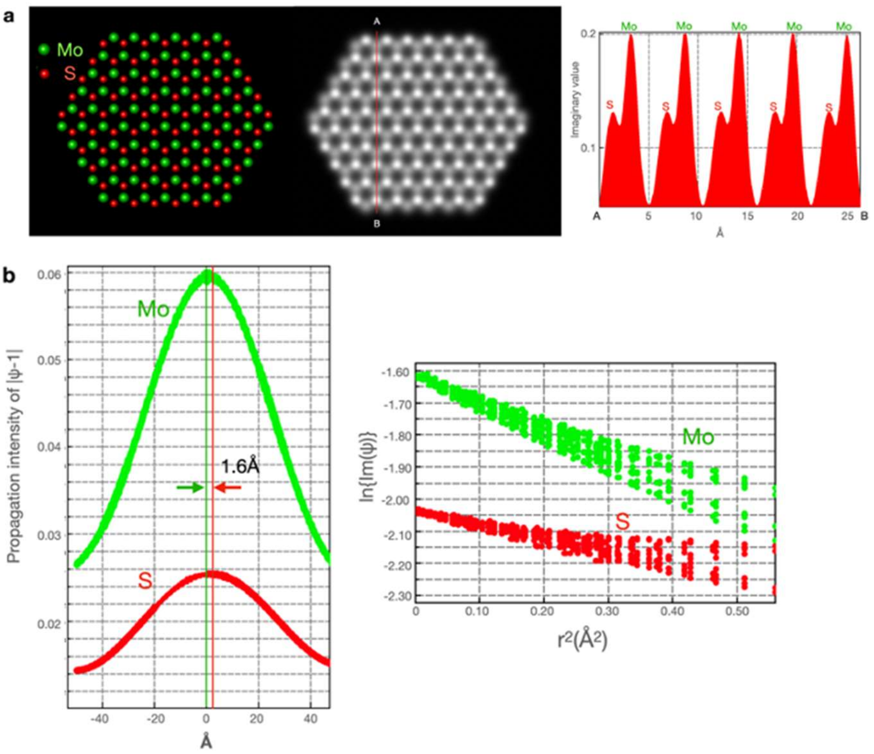

c

	R_{av}(Å)	θ (rad)	V' (V. Å)	V (V. $Å^3$)
Mo	0.55	0.20	170.5	161.9
S	0.48	0.128	109.12	78.94

Supplementary Figure 9. Exit wave simulation of a single-layer MoS_2 nanocrystal. The simulations were based on the multi-slice approach for calculating the projected potential, as implemented in the MacTempas software (www.totalresolution.com). **a**, Ball model of a MoS_2 nanocrystals with bulk-truncated $\{100\}$ edge terminations, its simulated exit wave imaginary part, and an intensity line-scan (distance in Å) through the latter. The modelled MoS_2 crystal structure adapts lattice parameters a = 3.16 Å, corresponding to S-S in-plane distance, and b = 1.5 Å corresponding to S-S interlayer distance along the c-axis. The simulations used Doyle and Turner's electron scattering factors[1] and the experimental parameters for the primary electron energy (50 keV) and information limit (g_{max} = 0.75 $Å^{-1}$). Moreover, to include phonon scattering, the frozen phonon method should in principle be adopted in which multi-slice calculations are repeated for a large number

of atom positions that are calculated with phonon calculation programs[2]. However, in the present case of a thin object, where scattering is linear, imaging commutes with the time averaging such that the time-dependent the atom positions can be represented by a DW factor implemented in a multi-slice code. Although earlier simulations used a constant DW for Mo and S [3], the element-specificity of atom vibrations is included here by varying the DW factor to quantitatively match simulated and measured phase peak values of the 1Mo and 2S atomic columns. Specifically, the <100> line-scan across the simulated exit wave imaginary part shows values of 0.20 and 0.13 for 1Mo and 2S atom columns, respectively, using DW factors of 30 $Å^2$ (Mo) and 60 $Å^2$ (S). These simulated peak values resemble the bulk *V'* measurements in Supplementary Fig. 5 of 120-160 eVÅ and 80-110 eVÅ, corresponding to phase values of up to ~ 0.197 and ~ 0.135 for 1Mo and 2S, respectively, since the phase θ is $\sigma V'$ and σ = 0.00123 $eV^{-1}Å^{-1}$at 50 keV. **b**, The simulated exit wave is analyzed following the exact same procedure as for the experimental exit waves (Methods). Specifically, one 1Mo and one 2S column are analyzed as all columns have similar appearance (e.g. line-scan in **a**). (left) The propagation of intensities of $|\Psi\text{-}1|$ captures the 1.6 Å difference in the *z*-height of the 1Mo and 2S columns consistent with Supplementary Fig. 3. (right) Plot of $\ln\{<\mathrm{Im}(\Psi(\mathbf{r}))>\}$ versus r^2 shows peak profiles that are more symmetric than the experimental case (Supplementary Fig. 7). **c**, The single atom column analysis of the simulated exit wave results in (i) R_{av} of about 50 pm, comparable to Supplementary Fig. 6, (ii) a peak value Θ and *V'* resembling Supplementary Fig. 5 as mentioned above, and (iii) integrated potential values, *V*, in agreement with Supplementary Table 1. The better agreement between experiment and simulations for *V* values as compared for *V'* is expected due to the counting statistics. Thus, the simulation of a single-layer MoS_2 exit wave with element-specific Debye-Waller factors can rationalize the experimentally determined parameters of the present model for the exit wave.

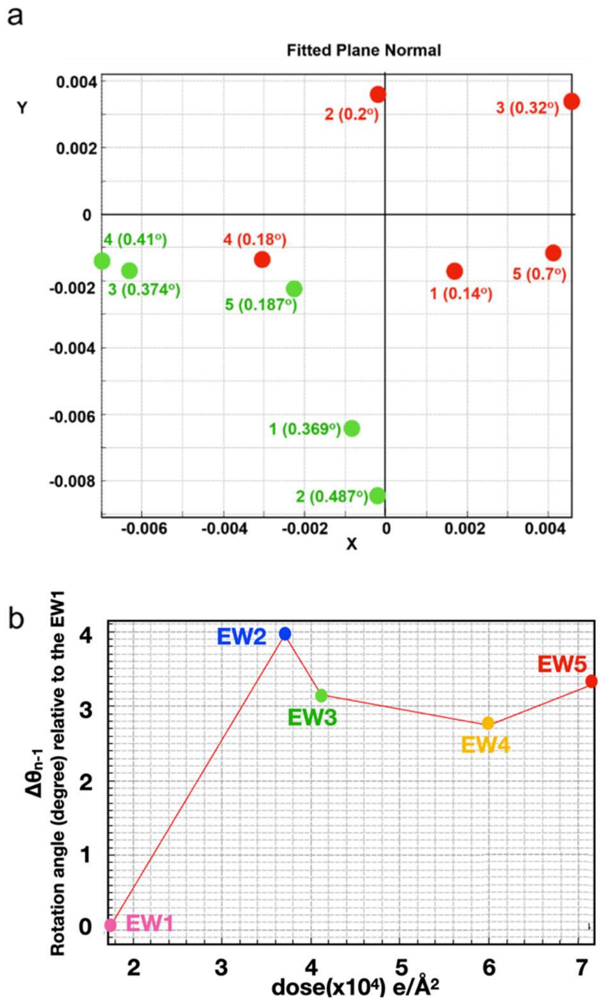

Supplementary Figure 10. Three-dimensional orientation of the nanocrystal. a, The tilt angle of the S and Mo basal planes for EW1-5 (denoted 1-5) is determined as the angle between the basal plane normal and electron beam direction. The basal plane normal were obtained by fitting 3D coordinates of the S and Mo lattices, respectively. The green dots show the Mo basal plane normal projected onto the image plane and the associated tilt angle. The red dots show the S basal plane normal projected onto the image plane and the associated tilt angle. The tilt direction of Mo plane is always in the third quant while the tilt direction of S plane varies among the first, the second and the fourth quantum. **b,** Rotation angle of the Co-Mo-S nanocrystal in the successive exit waves relative to EW1. Such relatively small collective motion of all atoms in the nanocrystal probably stems from residual thermal drift or electron-excitation of the sample. In Fig. 3, the nanocrystal is shown in same projections only.

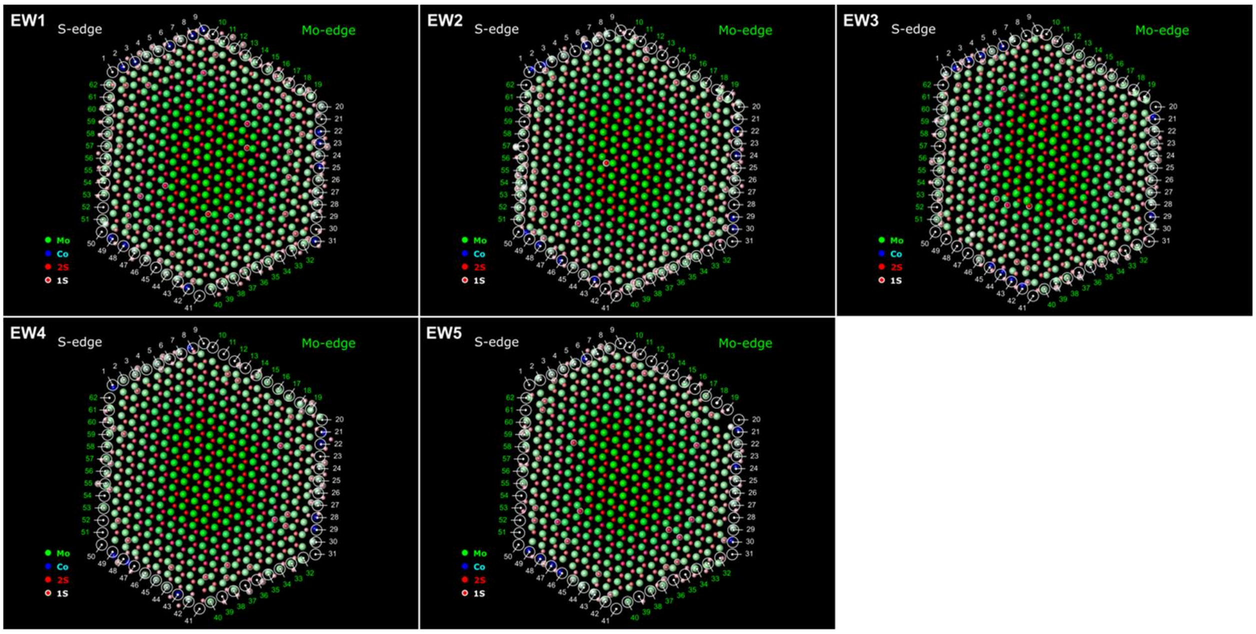

Supplementary Figure 11. Edge site analysis. The edge sites included in the analysis of the terminating metal atoms and their average S coordination number in Fig. 4c, d is indicated by the superimposed circles in the 3D atom-dynamic images of EW1-5.

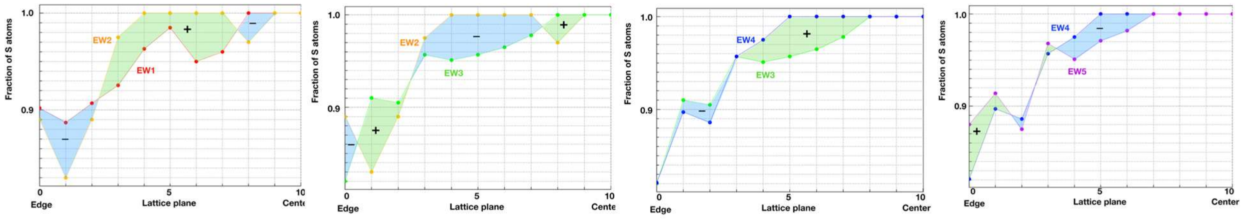

Supplementary Figure 12. Sulfur vacancy dynamics. The plot shows the sulfur vacancy dynamics deduced from the 3D atomic-resolution images (Fig. 3). The fraction of 1S atomic columns is defined as the number of S atoms relative to two times the number of S lattice sites. In the figure, the fraction of 1S columns is calculated within each radial lattice plane and plotted versus distance from the edge toward the center of the nanocrystal. The fraction of 1S columns is compared with that of the following exit wave. The blue zone shows reduction of 1S columns and the green zone shows increment of 1S columns in successive exit waves.

Supplementary Tables

EW1	X_o (eV•Å^3)	σ (eV•Å^3)
Mo	178.17	48.59
Co	57.97	23.70
2S	63.94	25.58
1S	34.95	11.94

EW2	X_o (eV•Å^3)	σ (eV•Å^3)
Mo	155.33	35.80
Co	52.00	7.67
2S	67.35	25.58
1S	32.40	15.77

EW3	X_o (eV•Å^3)	σ (eV•Å^3)
Mo	192.33	63.94
Co	49.87	32.82
2S	85.25	51.15
1S	35.80	17.05

EW4	X_o (eV•Å^3)	σ (eV•Å^3)
Mo	192.24	59.67
Co	42.63	21.31
2S	76.73	42.62
1S	34.10	12.79

EW5	X_o (eV•Å^3)	σ (eV•Å^3)
Mo	152.60	42.62
Co	59.67	12.79
2S	76.73	44.33
1S	34.10	13.64

Supplementary Table 1. The peak positions, x_o, and standard deviations, σ, for the fitted Gaussians to the *V* histograms in Supplementary Fig. 8 for the assigned 1Mo, 1Co, 2S and 1S atomic columns, respectively.

	EW1	EW2	EW3	EW4	EW5
Center	(-0.1099, -0.242323)	(0.025, -0.3429)	(-0.006, -0.2885)	(-0.072, -0.232)	(0.097, -0.0855)
Radius	1.1474	1.0381	1.066	1.1162	0.9087

Supplementary Table 2. Center and radius of circles fitted to the Argand plots (Supplementary Fig. 4).

EW1	V/πR²	V
Mo	329	326
Co	12	15
2S	614	626
1S	48	42

EW2	V/πR²	V
Mo	322	326
Co	11	7
2S	594	594
1S	39	39

EW3	V/πR²	V
Mo	323	327
Co	10	6
2S	592	592
1S	43	43

EW4	V/πR²	V
Mo	322	326
Co	9	5
2S	574	562
1S	35	41

EW5	V/πR²	V
Mo	316	319
Co	8	5
2S	564	550
1S	36	43

Supplementary Table 3. The number of 1Mo, 1Co, 2S and 1S columns in the tomograms determined from EW1-5 using $V/(\pi R^2)$ and V, respectively. The ratio of sulfur atoms (2*2S + 1*1S) and metal atoms (Mo + Co) is ca 1.9, which is slightly below the stoichiometric value of 2 for bulk MoS_2.

Supplementary Methods

Outline

The exit waves are obtained by focal series reconstruction and contain all the information that is present in the whole focal image series. The next step is then to use all the information that is present in the exit wave to determine the atomic structure of the object in a quantitative way with the highest precision. For this purpose, a simple analytical model is needed for the exit wave to enable model-based fitting with the experimental exit wave. Such a model must include the dynamical electron interaction within the sample with a minimal number of parameters and must also be valid up to a realistic sample thickness.

The present model considers samples in a zone axis orientation. Hereby, electrons are trapped in the atomic columns parallel to the viewing direction. The corresponding exit wave of an atomic column reveals the projected position of the column as well as a fingerprint of the mass-thickness of the column. Based on the channeling theory[4], an analytical model is derived for the exit wave of an atom column that is valid up to a larger depth than the usual phase object approximation. The method also includes the atomic vibrations and the microscope aberrations. As a result, the analytical approach becomes very robust and simple to implement.

Electron channeling

Channeling theory describes how the electron is trapped in an atomic column oriented parallel to the electron beam [4,5]. Within this theory, the electron wave Ψ inside the atom column is expanded as function of depth t in eigenstates Φ_n and eigenenergies E_n of the projected column potential:

$\Psi(\mathbf{r}, t) = \sum_n C_n \Phi_n(\mathbf{r}) \exp(iE_n t)$ (1)

with **r** the vector in the 2D plane orthogonal to the electron beam direction.

In case of a plane wave incident beam, [4] showed that for up to third order in $E_n t$

$\mathrm{Im}(\Psi(\mathbf{r}, t)) = \sum_n C_n \Phi_n(\mathbf{r})((E_n t)) = \sigma V_p(\mathbf{r})$ (2)

with $V_p(\mathbf{r})$ the projected electrostatic potential of the atom column (in units eV• Å), and $\sigma = \pi/(\lambda E)$ the interaction constant with E the primary energy of the electron and λ the electron wavelength.

The projected electrostatic potential $V_p(\mathbf{r})$ can be integrated numerically over **r** simply by adding the imaginary pixel values divided by σ. This integral then yields the 3D integral over the 3D electrostatic atom potential and is referred to as *V* (in units of eV·Å^3).

This integral can be calculated analytically using the Yukawa model for the 3D atom potential of an atom

$V_Y(\mathbf{R}) = kZe(1/R)\exp(-R/a)$ (3)

with **R** the position vector in three-dimensional space, k Coulomb's constant, *Z* the atomic number, e the electron charge and *a* the atom radius. The integrated $V_Y(\mathbf{R})$ over 3D becomes

$\int V_Y(\mathbf{R})d\mathbf{R} = kZe^2a^2$ (4)

Let *V* be the 2D integral of the imaginary part of the exit wave of the column or the sum of the imaginary values of the pixels, i.e.

$V = 1/\sigma\{\int \mathrm{Im}(\Psi(\mathbf{r}))d\mathbf{r}\}$ (5)

Hence,

$V = \int(V_p(\mathbf{r}))\, d\mathbf{r} = kZe^2a^2$ (6)

For the exit wave, this yields

$\mathrm{Im}\,(\Psi(\mathbf{r},t)) = kZe^2a^2V(\mathbf{r}) = \alpha Za^2V(\mathbf{r})$ (7)

The constant $\alpha = ke^2$ is the same for all atoms and $V(\mathbf{r})$ is a normalized function ($\int V(\mathbf{r})d\mathbf{r} = 1$) peaked at $r = 0$. Typical values for the atom radius a are 145, 135 and 100 pm, respectively, for Mo, Co and S. The advantage of this expression is that the first order term in the expansion of $\Psi(\mathbf{r},t)$ is imaginary whereas the second term is real-valued and both terms can be retrieved independently from the reconstructed exit wave. Furthermore, as shown in [4], the phase of the channeling wave is linear up to very large thicknesses. That is, the phase grating approximation

$\Psi(\mathbf{r}) = \exp(i\sigma V_p(\mathbf{r}))$ (8)

can be expanded up to third order as

$\Psi(\mathbf{r}) = \exp(i\sigma V_p(\mathbf{r})) = i\sigma V_p(\mathbf{r}) - (1/2)(\sigma V_p(\mathbf{r}))^2 - i(1/6)(\sigma V_p(\mathbf{r}))^3 + \ldots$ (9)

Thus, the imaginary terms yield

$\mathrm{Im}(\Psi(\mathbf{r})) = \sigma V_p(\mathbf{r}) - (1/6)(\sigma V_p(\mathbf{r}))^3$ (10)

showing that Im(Ψ) is linear in the phase $\varphi = \sigma V_p(\mathbf{r})$ with an error $\Phi^3/6$. A phase change of 0.75 mrad and an associated error of about 7% corresponds to about 2 Au atoms or 5 Cu atoms, corresponding to a Z ~ 140 according, Fig. 3 of [4] . Alternatively accepting a phase change of 1 mrad with an error of up to 16% corresponds to 3 Au atoms (total Z of 180) or 7 Cu atoms (total Z of 210). Thus, the present analysis should be limited to columns with a total $Z < 200$, which is the case for the MoS_2 sample under study. If the resolution of the EM is sufficient to resolve neighboring columns in 2D the linear term $\alpha Z a^2 V(\mathbf{r})$ provides information of the 2D position of the atom column and the parameter αZ that can be fitted ("refined") as independent parameters. At this stage, it is noted that the phase of an exit wave can only be determined modulo a constant phase φ and the amplitude can only be determined apart from a constant undetermined complex factor.

In case of a nanoparticle (such as MoS_2) one can often also determine the vacuum wave $\Psi_V(\mathbf{r})$ outside the particle where $V(\mathbf{r}) = 0$ so that the reconstructed exit wave $\Psi_R(\mathbf{r},t)$ can be normalized ("flatfielded") yielding

$\Psi_N(\mathbf{r}) = (\Psi_R(\mathbf{r},t) - \Psi_V(\mathbf{r}))/ \Psi_V(\mathbf{r}) = i\alpha Z a^2 V(\mathbf{r})$ (11)

And using

$\int V(\mathbf{r})d\mathbf{r} = 1$ (12)

We finally have

$\int \mathrm{Im}(\Psi_N(\mathbf{r}))\, d\mathbf{r} = \alpha Z a^2$ (13)

In conclusion, for a very thin object consisting only of a very small number of atoms in the direction of the incident beam, the total volume (integral) V of the imaginary part of the exit wave of the atom column is proportional to the total atom number Z of the atoms of the column. This conclusion

remains valid even if the exit wave is further blurred by coherent and incoherent microscopic aberrations and by the thermal vibration of the atoms.

It is well-known that the focal series reconstruction method cannot retrieve accurately the low spatial frequencies. Thus, it is possible that a small phase ramp remains in the exit wave which may yield a relatively large phase bias over the sample. In that case it is better to measure the vacuum wave through empty (interstitial) sites of the sample as has been done in[6].

Microscopic aberrations

Coherent aberrations

The coherent aberrations such as spherical aberration and defocus are corrected in standard focal series reconstruction algorithms. But since the exit-face of the sample is not necessarily flat the distance between the exit-face of a column and the plane of the reconstructed exit wave can vary from column to column. As shown in [6] this distance can be determined and corrected with sub-Angstrom precision. Thus, we can determine the position of the exit wave of a column projected along the axis of incidence with sub-Angstrom precision.

Incoherent aberrations

The present model is linear, and the sample is thin (Supplementary Fig. 4). An important advantage of a linear interaction model is that the incoherent aberrations of the EM such as temporal and spatial incoherence can be described by envelope functions in Fourier space and thus by convolutions in real space [5]. Thus, in the linear model, it is a very good approximation to describe the aberrations by the convolution with the normalized point spread function P(**r**) of the electron microscope. Thus, the aberrated exit wave is

$$\Psi_N(\mathbf{r}) \otimes P(\mathbf{r}) = i\alpha Z a^2 (V(\mathbf{r}) \otimes P(\mathbf{r})) \qquad (14)$$

The $\otimes$ is a convolution operator.

Thermal atom motion

The recording time of a high-resolution TEM image (order of 1 second) is much larger than the period of the thermal vibration of an atom. Thus, during the recording the atom position is statistically distributed with a normalized probability density function b(**r**). Under linear imaging conditions, the image of a displaced atom is the same as the displaced image of the atom so that the intensity of the atom is convoluted ("blurred") with the function b(**r**). As shown in [7], the exit wave of a thin object is a kind of weighted linear average of the images of the focal series so that this convolution is also present in the linear part of the exit wave yielding

$<\Psi_N(\mathbf{r})> = i\alpha Z a^2 (V_{eff}(\mathbf{r}))$ (15)

With < > denoting time averaging, $V_{eff}(\mathbf{r}) = V(\mathbf{r}) \otimes P(\mathbf{r}) \otimes b(\mathbf{r})$ and V(**r**), P(**r**) and b(**r**) are normalized functions, which finally yields

$\int \mathrm{Im}(<\Psi_N(\mathbf{r})>)\, d\mathbf{r} = V$ (16)

With $V = \alpha Z a^2$ (17)

Thus, $\alpha Z a^2$ can be determined by calculating the volume V under the imaginary peak value $\Psi_N(\mathbf{r})$. In practice, this can be done by summing all the imaginary pixel values around the atom. Since all these pixels are used to calculate one parameter, the result can be accurate even in case of a low number of imaging electrons. Moreover, a detailed model for the blurring functions is not needed.

But, in order to determine Za^2 from αZa^2, the calibration factor α must be determined. If single atom sensitivity is reached, the measured values αZa^2 can be plotted for all columns of the sample in one histogram and peaks at the discrete values of Za^2 can be discriminated. With pre-knowledge of possible Za^2 values of the atoms in the sample, the ultimate Z calibration is then obtained [8]. However, there is still a limitation. The normalization requirements only hold if all the pixels are captured.

Overlapping atom waves

If the total blurred peaked wave $V_{eff}(\mathbf{r})$ is large so that the peaks of neighboring atoms overlap, the pixels of the respective atoms cannot unambiguously be identified and hence the value V cannot be determined with sufficient accuracy. In order to handle this situation, the shape of the blurring

function $V_{eff}(\mathbf{r})$ can be modelled by for instance a Gaussian with radius R. This extra parameter R can be determined from the pixels in the non-overlapping area and the integral under this peak can be calculated analytically. The model for such a peak f(r) is the Gaussian

$$f(r) = (V/(\pi R^2))\exp(-r^2/R^2) \quad (18)$$

The peak value of f(r) at r = 0 is $V' = V/(\pi R^2)$, in units of eV•Å, which is actually the projected potential scaled by the averaged area over which the atoms are smeared out. $V'\exp(-r^2/R^2)$ can be determined as function of r^2 based on the pixel values of an atomic column. Calculating the natural logarithm yields

$$\ln f(r) = \ln (V/(\pi R^2)*\exp(-r^2/R^2)) = \ln(V/(\pi R^2)) - r^2/R^2 \quad (19)$$

Since this function is linear in r^2, linear regression enables a determination of the intercept

$$\ln (V/(\pi R^2)) = \ln V - \ln(\pi R^2) \quad (20)$$

and the slope $1/R^2$.

V and R and their respective standard deviations can in principle be obtained by linear regression and from $V = \alpha Za^2$ the Z value can eventually be derived. Also, the radius R can give extra information about the thermal blurring of the atom.

Since the present images are very noisy, averaging is needed before calculating the logarithm and before the linear regression. Assuming that the peak is azimuthally symmetric (isotropic), the Gaussian model contains only two parameters V and R. The situation is, however, more complicated if the atomic column is azimuthally blurred. In that case, R can be represented by an averaged atomic column radius R_{av}. A simple approach to take the anisotropic shape profile into account is to determine R_{av} as the average of R_{max} and R_{min} being the longest and shortest radius of an anisotropic atomic column, respectively. The determination of R_{max} and R_{min} is given in the method section.

Supplementary References

1. Doyle, P. A. & Turner, P. S. Relativistic Hartree–Fock X-ray and electron scattering factors. *Acta Crystallogr. Sect. A* **A24**, 390 (1968).
2. Van Dyck, D. Is the frozen phonon model adequate to describe inelastic phonon scattering? *Ultramicroscopy* **109**, 667 (2009).
3. Kisielowski, C. *et al.* Imaging MoS_2 nanocatalysts with single-atom sensitivity. *Angew. Chem. Int. Ed.* **49**, 2708–2710 (2010).
4. Van Dyck, D. & Op De Beeck, M. A simple intuitive theory for electron diffraction. *Ultramicroscopy* **64**, 99–107 (1996).
5. Van Dyck, D. Atomic resolution electron microscopy. in *Handbook of Nanoscopy* (eds. Tendeloo, G. Van, Dyck, D. Van & Pennycook, S. J.) 45–79 (Wiley-VCH, 2012).
6. Chen, F. R., Kisielowski, C. & Van Dyck, D. 3D reconstruction of nanocrystalline particles from a single projection. *Micron* **68**, 59–65 (2015).
7. Op De Beeck, M., Van Dyck, D. & Coene, W. Wave function reconstruction in HRTEM: The parabola method. *Ultramicroscopy* **64**, 167–183 (1996).
8. Chen, F. R., Van Dyck, D. & Kisielowski, C. In-line three-dimensional holography of nanocrystalline objects at atomic resolution. *Nat. Commun.* **7**, 10603 (2016).

Supplementary Movie 1.

The successive 3D atom-dynamic images based on EW1-5 (Supplementary Fig. 3) reveal marked atom displacements at the edge of the Co-Mo-S nanocrystal.
