# Supplementary material for: Probing atom dynamics of excited Co-Mo-S nanocrystals in 3D
Source: Nat Commun. 2021 Aug 18;12:5007. doi: 10.1038/s41467-021-24857-4 (PMC8373969; doi:10.1038/s41467-021-24857-4)
Supplement: Supplementary file 2 — Description of Additional Supplementary Files [file 41467_2021_24857_MOESM2_ESM.docx]

**Description of Additional Supplementary Files**

**Supplementary Movie 1:**

The successive 3D atom-dynamic images based on EW1-5 (Supplementary Fig. 3) reveals marked atom displacements at the edge of the Co-Mo-S nanocrystal.
